# Clinical exome analysis and targeted gene repair of the c.1354dupT variant in iPSC lines from patients with *PROM1*-related retinopathies exhibiting diverse phenotypes

**DOI:** 10.1186/s13287-024-03804-2

**Published:** 2024-07-02

**Authors:** Kevin Puertas-Neyra, Rosa M. Coco-Martin, Leticia A. Hernandez-Rodriguez, Dino Gobelli, Yenisey Garcia-Ferrer, Raicel Palma-Vecino, Juan José Tellería, Maria Simarro, Miguel A. de la Fuente, Ivan Fernandez-Bueno

**Affiliations:** 1 Instituto Universitario de Oftalmobiología Aplicada (IOBA), Universidad de Valladolid, Valladolid, Spain; 2 Redes de Investigación Cooperativa Orientadas a Resultados en Salud (RICORS-REI), Inflamación E Inmunopatologia de Organos y Sistemas, Instituto de Salud Carlos III, Valladolid, Spain; 3 Centro en Red de Medicina Regenerativa, y Terapia Celular de Castilla y León, Valladolid, Spain; 4 Unidad de Excelencia Instituto de Biomedicina y Genética Molecular (IBGM), Universidad de Valladolid y Consejo Superior de Investigaciones Científicas (CSIC), Valladolid, Spain; 5 Departamento de Biología Celular, Genética, Histología y Farmacología, Facultad de Medicina, Universidad de Valladolid, Valladolid, Spain

**Keywords:** iPSC, Retinal diseases, PROM1 gene, CD133, Retinitis pigmentosa, Cone-rod dystrophy, Stargardt’s type 4 disease

## Abstract

**Background:**

Inherited retinal dystrophies (IRD) are one of the main causes of incurable blindness worldwide. IRD are caused by mutations in genes that encode essential proteins for the retina, leading to photoreceptor degeneration and loss of visual function. IRD generates an enormous global financial burden due to the lack of understanding of a significant part of its pathophysiology, molecular diagnosis, and the near absence of non-palliative treatment options. Patient-derived induced pluripotent stem cells (iPSC) for IRD seem to be an excellent option for addressing these questions, serving as exceptional tools for in-depth studies of IRD pathophysiology and testing new therapeutic approaches.

**Methods:**

From a cohort of 8 patients with *PROM1*-related IRD, we identified 3 patients carrying the same variant (c.1354dupT) but expressing three different IRD phenotypes: Cone and rod dystrophy (CORD), Retinitis pigmentosa (RP), and Stargardt disease type 4 (STGD4). These three target patients, along with one healthy relative from each, underwent comprehensive ophthalmic examinations and their genetic panel study was expanded through clinical exome sequencing (CES). Subsequently, non-integrative patient-derived iPSC were generated and fully characterized. Correction of the c.1354dupT mutation was performed using CRISPR/Cas9, and the genetic restoration of the *PROM1* gene was confirmed through flow cytometry and western blotting in the patient-derived iPSC lines.

**Results:**

CES revealed that 2 target patients with the c.1354dupT mutation presented monoallelic variants in genes associated with the complement system or photoreceptor differentiation and peroxisome biogenesis disorders, respectively. The pluripotency and functionality of the patient-derived iPSC lines were confirmed, and the correction of the target mutation fully restored the capability of encoding Prominin-1 (CD133) in the genetically repaired patient-derived iPSC lines.

**Conclusions:**

The c.1354dupT mutation in the *PROM1* gene is associated to three distinct AR phenotypes of IRD. This pleotropic effect might be related to the influence of monoallelic variants in other genes associated with retinal dystrophies. However, further evidence needs to be provided. Future experiments should include gene-edited patient-derived iPSC due to its potential as disease modelling tools to elucidate this matter in question.

**Graphical Abstract:**

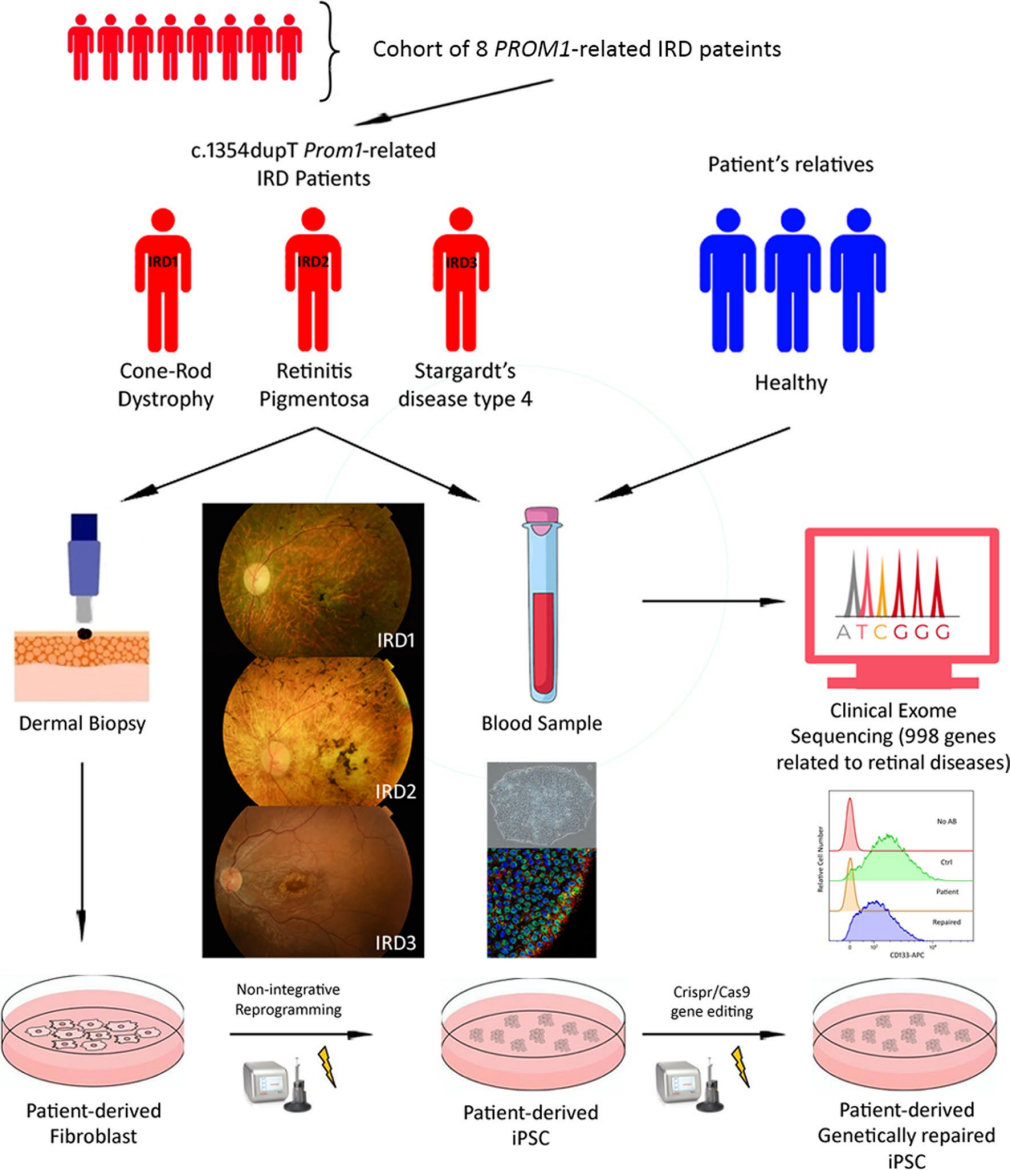

**Supplementary Information:**

The online version contains supplementary material available at 10.1186/s13287-024-03804-2.

## Background

Inherited retinal dystrophies (IRD) are a group of rare Mendelian inheritance diseases caused by mutations in genes that encode essential proteins for various retinal cell processes, usually leading to visual loss due to progressive photoreceptor degeneration [1, 2]. IRD are the main cause of visual impairment in the working age group of 16 to 65 years and the second most common reason among children, affecting their quality of life and imposing an enormous financial burden globally [3, 4].

Progressive IRD can be clinically subclassified as Retinitis Pigmentosa (RP), which is the most prevalent form and primarily affects rods; Progressive Cone Dystrophy (PCD), primarily affecting cones, although it frequently evolves to Cone-Rod Dystrophy (CORD); and Macular Dystrophy (MD), affecting both rods and cones in the macular region. Inheritance patterns include autosomal dominant (AD), autosomal recessive (AR), and X-linked (XL) Inheritance [5–7].

Various clinical presentations of inherited retinal diseases (IRD) may arise from distinct variants within the same gene [8–11], while specific mutations in a single gene can result in different IRD phenotypes [12–16]. Due to the significant phenotypic and genotypic heterogeneity, molecular diagnosis based on Next Generation Sequencing (NGS) methodologies is deemed essential for IRD diagnosis [17, 18]. NGS techniques, including targeted panels or Clinical Exome Sequencing (CES) have shown considerable utility in accurately determining genotype–phenotype correlations in IRD patients [18–21].

*PROM1*-related retinopathies can manifest as pan-retinal phenotypes, as in Retinitis Pigmentosa type 41 (RP type 41, OMIM 268000) and Cone-Rod Dystrophy (CORD, OMIM 604116 or 120,970); or as macular dystrophies such as Stargardt's disease type 4 (STGD4, OMIM 248200) [22, 23]. Additionally, some specific single mutations in the *PROM1* gene have been associated with both AD and AR forms of the disease, commonly AD forms tend to be of later onset and AR earlier [8, 23, 24]. Even within the same family, it has been reported substantial differences in the severity and extension of retinal degeneration. This clinical heterogeneity may be due to the influence of modifier genes, epigenetic and environmental factors [25].

*PROM1* encodes the 5-domains transmembrane glycoprotein Prominin-1 (CD133) [26]. Historically recognised as stem cell marker, plays a crucial role in fundamental cellular processes, including self-renewal, metabolism or differentiation [27]. In the retina, prominin-1 is expressed at the base of the photoreceptors’ outer segments (OS). It is fundamental for the morphogenesis and structure of the OS membranes due to its interaction with the protocadherin-21, actin filaments, and myosin II, and it also plays an important role in the regulation of calcium-dependent chlorine channels [28, 29]. Likewise, it has significant implication in the regulation of autophagy in the retinal pigmented epithelium (RPE), and its deficiency increases apoptosis in retinal glial cells [30, 31].

There is currently no treatment available for IRD, apart from the unique additive therapy for biallelic mutations in the *RPE65* gene (Luxturna, *voretigene neparvovec-rzyl*) established in 2017 [32]. However, gene therapy for IRD is an active area of research, and some clinical trials have shown encouraging results, such as those conducted on *RPGR* or *CNGA3* genes [33, 34]. Other gene therapy clinical trials have been paused due to various issues that need to be addressed, as seen in the case of *CHM* gene [35]. The challenge lies in the limitation of specific gene therapy, making it difficult to correct the more than 300 genes associated with IRD [36]. To date, there are not potential therapeutic alternatives for patients in advanced stages of retinal degeneration other than stem cell therapy and optogenetics [37]. In this regard, patient-derived induced pluripotent stem cells (iPSC) appear to be a promising option for addressing these challenges.

iPSC are obtained by reprogramming somatic cells through the introduction of a set of pluripotency transcription factors, originally: Oct3/4, Sox2, c-Myc, and Klf4 [38]. They provide an accessible and abundant source of cells potentially capable of differentiating into retinal cells such as RPE and photoreceptors, without the ethical concerns associated to Embryonic Stem Cells (ESC) [39]. Patient-derived iPSC are valuable tools for in vitro disease modelling (disease-in-a-dish) and drug screening for retinal diseases [40]. In addition, iPSC with corrected mutations using CRISPR/Cas9 editing can be used to study gene function or generate cell therapy products as an alternative treatment to replace the degenerated retina in advanced disease [41–44]. At present, only two *PROM1*-related iPSC lines, both associated with the same mutation c.619G > T (p.E207X), have been reported [45].

Upon analysing a cohort of eight *PROM1*-related IRD patients we identified the pleiotropic effect of the IRD variant c.1354dupT (p.Tyr452Leufs*13) in homozygosity with AR inheritance. This mutation was linked to CORD, RP, and STGD4 in three different patients from our series. Therefore, the aim of this study was to elucidate the multi-phenotypic effect of the target mutation by expanding the patient’s genetic panel through CES, obtaining patient-derived iPSC, and genetically repairing those iPSC to study this mutation in vitro as an initial step towards designing new treatment options for these patients.

## Materials and methods

### Aim, design, and setting

To elucidate the pleiotropic effects of the IRD variant c.1354dupT (p.Tyr452Leufs*13) located in exon 13 of the *PROM1* gene, by identifying associated modifier genes, and to create gene-edited patient-derived iPSC. The study was structured as a transversal study and experimental research. It was carried out at the Institute for Applied Ophthalmobiology (IOBA) and at the Institute of Biomedicine and Molecular Genetics (IBGM), both affiliated with the University of Valladolid.

### Patient selection and sampling

A series of eight patients previously diagnosed with *PROM1*-related IRD was revised (Table 1). They underwent clinical and genetic characterization, and three presenting distinct phenotypes associated with the homozygous c.1354dupT (p.Tyr452Leufs*13) mutation were initially selected for the experimental study. Furthermore, a first-degree relative of each of these target patients was included.

**Table 1:** Genetic background of identified patients carrying *PROM1*-related retinopathies

Patient	Phenotype	Gender	Age at inclusion (years)	Ethnicity	Allele 1			Allele 2			Zygosity	Inheritance mode
					Exon/Intron	Nucleotide	Protein	Exon/Intron	Nucleotide	Protein		
IRD 1	CORD	F	47	Caucasic	Exon 13	c.1354dupT	p.Tyr452Leufs*13	Exon 13	c.1354dupT	p.Tyr452Leufs*13	Homozygosity	AR
IRD 2	RP	F	54	Caucasic	Exon 13	c.1354dupT	p.Tyr452Leufs*13	Exon 13	c.1354dupT	p.Tyr452Leufs*13	Homozygosity	AR
IRD 3	STGD4	M	20	Caucasic	Exon 13	c.1354dupT	p.Tyr452Leufs*13	Exon 13	c.1354dupT	p.Tyr452Leufs*13	Homozygosity	AR
IRD 4	CORD	F	32	Caucasic	Exon 5	c.615 T > A	p.Tyr205*	Intron 20–21?	c.2240G > A	p.Trp747*	Compound heterozygosity	AR
IRD 5	CORD	M	41	Caucasic	Exon 8	c.869delG	p.Ser290IleFs*2	Exon 8	c.869delG	p.Ser290IleFs*2	Homozygosity	AR
IRD 6	STGD 4	M	41	African	Exon 10	c.1117C > T	p.Arg373Cys	–	–	–	Heterozygosity	AD
IRD 7	STGD 4	M	37	Latin	Exon 10	c.1117C > T	p.Arg373Cys	–	–	–	Heterozygosity	AD
IRD 8	CORD	F	64	Caucasic	Intron 17	c.1984-1G > T	r.(spl?)	Intron 21	c.2327A > T	p.Asp776Val	Compound heterozygosity	AR

IRD: inherited retinal dystrophy; CORD: cone-rod dystrophy; STGD4: Stargardt disease type 4; RP: retinitis pigmentosa; AD: autosomal dominant; AR: autosomal recessive, F: female; M: male

Peripheral blood samples were collected from the patients and their respective relatives for CES. Dermal biopsy samples were obtained from the three target patients and were used to generate iPSC.

### Ophthalmological examination

To confirm the patient’s phenotype and establish their pedigree patterns, a comprehensive anamnesis and ophthalmological examination were performed. Visual acuity (VA) was tested using an Early Treatment Diabetic Retinopathy Study panel and recorded as the logarithmic of minimum angle of resolution (logMAR) scale. Retinography and fundus autofluorescence (FAF) were obtained with the TRC 50DX Retinograph (TOPCON Europe Medical BV, Rotterdam, the Netherlands). Sweep-source optical coherence tomography (OCT) was gathered using the PLEX® Elite 9000 OCT (Carl Zeiss AC). Visual field (VF) testing was performed when fixation was possible with the Humphrey Field Analyzer (Carl Zeiss Meditec, Dublin, CA). A full field electroretinogram (ERG) was also performed according to the regularly updated standards of the International Society for Clinical Electrophysiology of Vision (ISCEV) [46].

### Clinical exome sequencing

To investigate the potential influence of other modifying genes that could account for the varied phenotypic expressions associated with the target mutation, we conducted clinical CES of three target patients and three healthy relatives. DNA was extracted from blood cells, the Roche exome capture library was prepared, and extensive CES was carried out on a NovaSeq 6000 S4 (Illumina, CA, USA) with 90 × coverage. Bioinformatic analysis of the exons and adjacent intronic regions was performed on a total of 998 genes linked to retinal diseases (Supplementary file 1). We concentrated on genes associated with IRD [47], Age-related Macular Disease (AMD) [48], and genes coding for the phenotype HP:000047, indicative of abnormal retinal morphology [49]. Subsequently, *in-silico* predictors were applied as filters, and the findings were validated by Sanger sequencing using the ABI Prism 3130xl genetic analyzer (Thermo Fisher, MA, USA). Only variants classified as pathogenic or likely pathogenic were considered for further analysis.

### Induced pluripotent stem cells generation

#### Fibroblast isolation

**Fig. 1:**
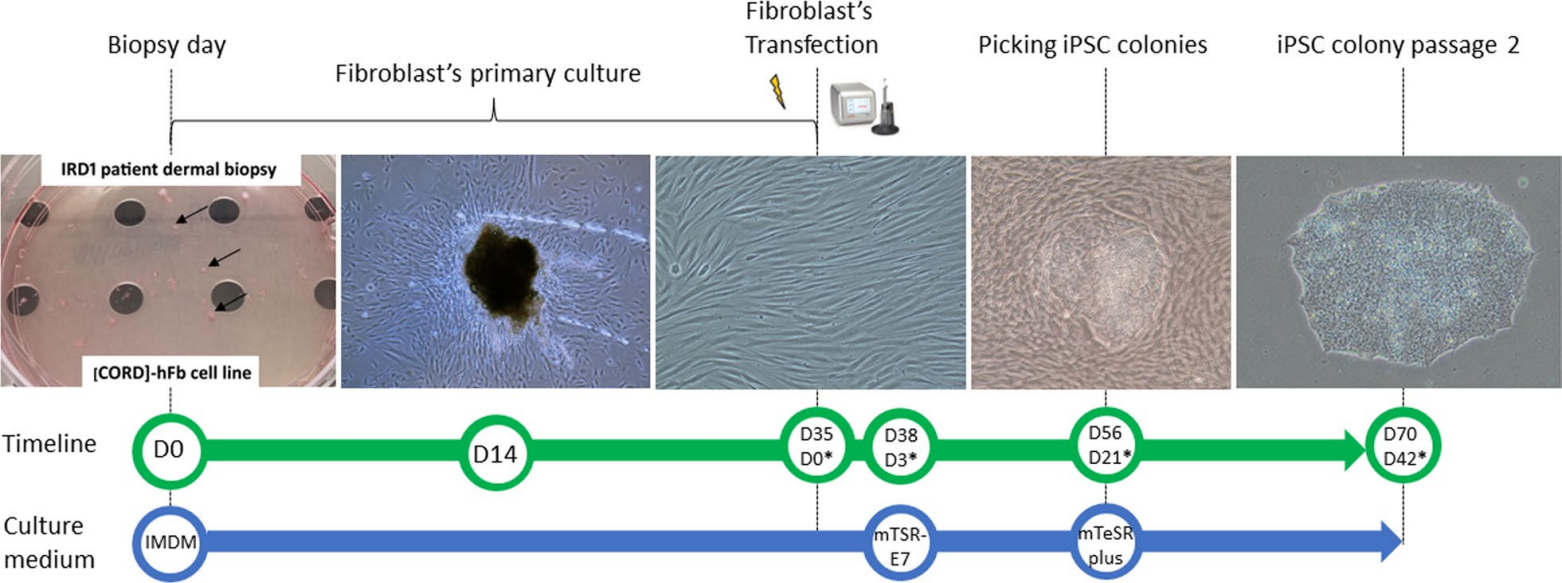
Generation of patient-derived iPSC from IRD1 patient. At D0, dermal biopsy is cut into small pieces, seeded in 100-mm culture dishes, and maintained with IMDM medium. At D14, outgrowth of fibroblast is noticeable from biopsy pieces. At D28, passage 2 patient-derived fibroblast are reprogrammed using the episomal vectors through electroporation. At D38, three days after electroporation, IMDM medium is changed to mTSR-E7 medium. At D56, three weeks after electroporation, iPSC colonies have emerged between fibroblasts. iPSC colonies are picked up manually and seeded in new culture dishes using the mTeSR plus medium. At D70, passage 2 iPSC colonies displaying compact colonies with distinct borders, well-defined edges, and large nuclei. Days after electroporation*

A dermal biopsy was performed at the patient’s cranial gluteal area, previously disinfected with 10% povidone iodine and rinsed with saline solution, under local anesthesia injected subcutaneously (1% lidocaine), using a 3-mm biopsy punch (Stiefel, Middlesex, UK). The biopsies were immersed in IMDM medium supplemented with 10% fetal bovine serum (FBS), 3% penicillin/streptomycin (P/S), 3X Amphotericin B and 0.1 µM 2-Mercaptoetanol (Gibco, Invitrogen, Paisley, UK. Cat# 11,510,596, 26,140,079, 11,548,876, 15,290,018, 31,350,010). Biopsies were then cut into small pieces (small as possible, approximately 0.5 × 0.5 mm) and cultured in collagen-coated (Sigma-Aldrich, Saint Louis, USA. Cat# C3867-1VL) 100-mm culture dishes (Thermo Fisher, MA, USA. Cat# A30907) using “fibroblast medium”, composed by IMDM supplemented with 10% FBS, 1X P/S and 1X Amphotericin B and 0.1 µM 2-Mercaptoetanol (Gibco, Invitrogen, Paisley, UK. Cat# 11,510,596, 26,140,079, 11,548,876, 15,290,018, 31,350,010) under standard conditions (37Cº, 5% CO2 atmosphere). The fibroblast medium was refreshed every two days. Biopsies pieces were maintained in culture until fibroblasts outgrowth was observed, approximately at day 10 to 14 (Fig. 1).

#### Non-integrative fibroblast reprogramming

The patient’s fibroblasts were reprogrammed using The Epi5 Episomal iPSC Reprogramming Kit (Thermo Fisher, MA, USA. Cat# A15960) containing the reprogramming vectors pCE-hOCT3/4 (*OCT4* gene), pCE-hSK (*Sox2* and *KlF4* genes), pCE-hUL (*L-Myc* and *Lin28* genes) in Tube A, and the pCE-mP53DD (*mp53DD* gene), and pCXB-EBNA1 (*EBNA1* gene) in Tube B. Passage 2 fibroblast (150,000 cells) were electroporated with 1 µL of Tubes A and B, using the 100 µL pipette tip of the Neon Transfection System (Thermo Fisher, MA, USA. Cat# MPK5000) under the following pulse conditions: 1400 V; 20 ms; two pulses. The electroporated fibroblast were then seeded in Matrigel-coated (23 µg/cm^2^; Corning Life Sciences, NY, USA. Cat# 11,593,620) 6-well plates (Thermo Fisher Scientific, MA, USA. Cat# 11,337,694) using fibroblast medium, being changed every 24 h until day 3. Subsequently, the medium was replaced by the TeSR™-E7™ Medium (Stem Cells Technologies, Cambridge, UK. Cat# 05914) until iPSC colonies emerged, approximately 21 days. The iPSC clones were manually picked using a 22Gx2″ hypodermic needle (Terumo, Madrid, Spain), the Leica LED2500-TL3000 ERGO microscope (Thermo Fisher, MA, USA), and transferred with a p200 Pipette (Gilson PIPETMAN; Thermo Fisher Scientific, MA, USA. Cat# 1,232,613) to a Matrigel-coated (23 µg/cm^2^; Corning Life Sciences, NY, USA. Cat# 11,593,620) 6-well plate with mTeSR™ Plus medium supplemented with 10 µM Rock inhibitor Y-27632 (Stem Cells Technologies, Cambridge, UK. Cat# 100–0276, 72,304). iPSC were passaged as clumps using 0.5 mM EDTA (Thermo Fisher, MA, USA. Cat# 10,135,423) every 5–7 days, and frozen using freezing medium composed by 90% FBS (Thermo Fisher, MA, USA. Cat# 26,140,079) and 10% DMSO (Sigma-Aldrich, Saint Louis, USA. Cat# 34,869) (Fig. 1).

### Induced pluripotent stem cells (iPSC) characterization

#### Alkaline phosphatase staining

To demonstrate the characteristic upregulation of alkaline phosphatase (AP) activity in the generated iPSC lines, we performed the AP blue membrane substrate solution assay (Sigma-Aldrich, Saint Louis, USA. Cat# AB0300). On day 5, a single 6-well plate (Thermo Fisher Scientific, MA, USA. Cat# 11,337,694) was fixed with 4% paraformaldehyde (Panreac Quimica, Barcelona, Spain) for 1 min. The plate was then washed with phosphatase-buffered saline (PBS) prewarmed to 64 °C (Thermo Fisher, MA, USA. Cat# 10,010,023) and incubated for 20 min at the same temperature. Afterwards, 1.5 ml of a 1:1 mixture of kit solutions A and B was added, and the plate was incubated in the dark at room temperature (RT) for 10 min. Direct micrographs were obtained using a Nikon Eclipse TS100 inverted microscope (Nikon Instruments, NY, USA).

#### Immunocytochemical characterization

Immunocytochemical characterization was performed as previously described [50]. Briefly, iPSC lines were grown on Matrigel-coated (23 µg/cm^2^; Corning Life Sciences, NY, USA. Cat# 11,593,620) μ-Slide 8-well culture plates (Ibidi, München, Germany. Cat# 80,826) and fixed with 10% formalin solution (Sigma-Aldrich, Saint Louis, USA. Cat# HT501128). The plates were rinsed with Tris-buffered saline (TBS; Sigma-Aldrich, Saint Louis, USA. Cat# T5912) and blocked with a mixture of TBS, 0,3% Triton X-100 and 3% donkey serum, (Sigma-Aldrich, Saint Louis, USA Cat# X100, D9663) for 60 min. Primary antibodies associated with pluripotency markers (OCT4, SSEA3, SOX2, SSEA4, TRA1-60, NANOG, TRA-1–81) and germ layers markers (β-III Tubulin, α-1 Fetoprotein, α- Smooth Muscle Actin) were applied, followed by incubation with their respective species-specific secondary antibody. Nuclei were visualized by immunostaining with DAPI (4',6-diamidino-2-phenylindole, Invitrogen, CA, USA. Cat# 10,184,322). The specific conditions for the primary and secondary antibodies are detailed in Supplementary file 2.

All immunocytochemical analyses were carried out in triplicate for each experimental condition. Controls, were primary and/or secondary antibodies were omitted, were processed concurrently. Immunofluorescence micrographs were captured using a LEICA TCS SP8 LIGHTNING confocal microscope (Leica Microsystems, Hesse, Germany) and analysed with LEICA LAS AF software (Leica Microsystems, Hesse, Germany).

#### Expression of pluripotency factors and silencing of reprogramming vectors

To assess the expression levels of endogenous pluripotency factors, we conducted a gene-specific primer-based quantitative reverse transcription PCR (qRT-PCR) for the genes SOX2, OCT3/4, KLF4, LMYC, LIN28. On the other hand, to probe the absence of reprogramming vectors, we performed a dual assay: a copy number qPCR, using genomic DNA (gDNA) and targeting a region common to all reprogramming vectors (within the *EBNA1* gene), and a vector-specific primer-based qRT-PCR, using complementary DNA (cDNA), for each exogenous reprogramming factor delivered by the reprogramming vectors.

For qRT-PCR assays, RNA was extracted from the patient-derived iPSC lines at passage 6 using the Trizol Reagent (Invitrogen, CA, USA. Cat# 15,596,026) according to the manufacturer’s protocol. The purity and concentration of the RNA were determined using a NanoDrop 2000 spectrophotometer (Thermo Fisher Scientific, MA, USA). cDNA synthesis was performed using the High-Capacity cDNA Reverse Transcription Kit (Applied Biosystems, CA, USA. Cat# 4,368,814), following the manufacturer’s guidelines.

For *EBNA1* copy number qPCR, gDNA extraction was performed using ethanol precipitation [51]. A standard curve was generated from serial dilutions of a vector contained in the Epi5 Episomal iPSC Reprogramming Kit (Thermo Fisher, MA, USA. Cat# A15960), pCE-hOCT3/4 (Addgene #41,813). The number of copies of *EBNA1* in each dilution was determined based on the length of the pCE-hOCT3/4 plasmid.

All qPCRs were carried out with SYBR Green PCR Master Mix (Applied Biosystems, CA, USA. Cat# 4,309,155) on the LightCycler 480 Instrument II System (Roche, Switzerland). The cycling conditions were as follows: an initial denaturation at 95 °C for 10 min, followed by 45 cycles of 95 °C for 15 s, 60 °C for 30 s, and 72 °C for 45 s, with a final extension at 72 °C for 5 min and a subsequent melting curve analysis. *GAPDH* served as the housekeeping gene for normalizing mRNA expression levels. The threshold cycle was determined for each reaction, and gene expression levels were quantified using the 2^−ΔΔCt^ method [51]. All qPCR assays were conducted in triplicate for each experimental condition.

Untransfected fibroblasts from IRD1 patient served as the negative control for the expression of pluripotency factors essay; while fibroblasts from IRD1, transfected with the same episomal vectors as the iPSC lines, 72 h post-transfection, served as positive control. Primers used in qRT-PCR essays for endogenous pluripotency factors (*gene* primers) and exogenous reprogramming vectors (*plasmid* primers) are listed in Supplementary file 3.

#### In vitro* differentiation into three germinal layers*

To assess the differentiation potential of patient-derived iPSC lines into cells from the three germ layers, three-dimensional iPSC aggregates known as embryoid bodies (EBs) were specifically induced to differentiate into cell types characteristic of each layer. iPSC were cultured to 80% confluence on Matrigel-coated (23 µg/cm^2^; Corning Life Sciences, NY, USA. Cat# 11,593,620) 60 mm culture plates (Thermo Fisher, MA, USA). They were then dissociated with 0,5 mM EDTA (Thermo Fisher, MA, USA. Cat# 10,135,423), resuspended in mTeSR™ Plus medium (Stem Cells Technologies, Cambridge, UK. Cat# 100–0276) and seeded into 96-well V-bottom ultra-low attachment plates (Sigma-Aldrich, Saint Louis, USA. Cat# 10,462,012), which were centrifuged to facilitate EB formation. The EBs were cultured in ultra-low attachment 60-mm plates (Corning Life Sciences, NY, USA. Cat# 3261) for 3 days, subsequently transferred to Matrigel coated (23 µg/cm^2^; Corning Life Sciences, NY, USA. Cat# 11,593,620) μ-Slide 8 well culture plates (Ibidi, München, Germany. Cat# 80,826) and maintained for two to three weeks with three distinct differentiation media: endoderm medium (DMEM supplemented with 20% FBS, 2 mM Glutamax™, 100 μM non-essential amino acids, 100 μM 2-Mercaptoetanol and 1X P/S, [Gibco, Invitrogen, Paisley, UK. Cat# 13,345,364, 26,140,079, 35,050,061, 11,140,050, 11,528,926, 11548876]), mesoderm medium (endoderm medium supplemented with 100 μM ascorbic acid [Sigma-Aldrich, Saint Louis, USA. Cat# V-038]), and ectoderm medium (50% DMEM F12, 50% neurobasal medium, 2 mM Glutamax™, 1X N2 supplement, 1X B27 supplement and 1X P/S [Gibco, Invitrogen, Paisley, UK. 11,320,033, 21,103,049, 35,050,061, 17,502,048, 17,504,044, 11548876]).

#### Short tandem repeat analysis

To verify the genetic concordance between the patient’s fibroblasts and their respective iPSC lines, DNA fingerprinting analysis was conducted. The amplification of short tandem repeat (STR) was performed through multiple PCR using the kit GenePrint® 10 System PCR Amplification Kit (Promega, Wisconsin, USA. Cat# B9510). STR analysis at 10 loci (CSF1PO, D13S317, D16S539, D21S11, D5S818, D7S820, TH01, TPOX, vWA, and Amelogenin for sex determination) were carried out in accordance with the ASN-0002 standard established by the American Tissue Culture Collection Standards Development Organization Workgroup for cell line authentication. The amplified samples were evaluated on a 3730 DNA Analyzer (Applied Biosystems. CA. USA) using the POP-7 polymer and the ILS600 size standard. The results were analysed using the GeneMapper® software (v4.1, Applied Biosystems).

#### Karyotyping

To assess the genomic integrity of the generated iPSC lines, a G-banded metaphase chromosome analysis with a resolution of 300–500 bands was conducted. The patient-derived iPSC colonies at 70% of confluence were incubated in KaryoMax colcemid (Thermo Fisher, MA, USA. Cat 15,212,012) at a final concentration of 0,1 μg/mL for 3 h to induce mitotic arrest during metaphase. The cells were then trypsinized, exposed to a prewarmed 37ºC hypotonic solution (KCl 75 mM) for 15 min and fixed with methanol/glacial acetic acid (3:1) solution. A total of 20 metaphases were analysed.

#### Target mutation sequencing

The target mutation (c.1354dupT) was sequenced in the iPSC lines. gDNA extraction was performed using ethanol precipitation [52]. The region of the *PROM1* gene containing the target mutation was amplified by conventional PCR. Subsequently, the PCR-amplified fragments were sequenced using Capillary Electrophoresis Sanger Sequencing on an ABI 3730xl DNA analyser (Thermo Fisher, MA, USA). The sequencing primer *PROM1seqFW* is listed in Supplementary file 3.

### Patient-derived iPSC genetic repair

#### gRNA designing

The *gRNA* were designed in silico using the CRISPOR web tool for genome editing [53], selected based on their proximity to the mutation and their specificity and efficiency scores. gRNAs were synthesized in vitro using the GeneArt™ Precision gRNA Synthesis Kit (Invitrogen, Paisley, UK. Cat# A29377) following manufacturer’s instructions. Four *gRNAs (40RE, 47FW, 66FW, 77FW)* and their corresponding repair oligonucleotides were generated.

**Fig. 2:**
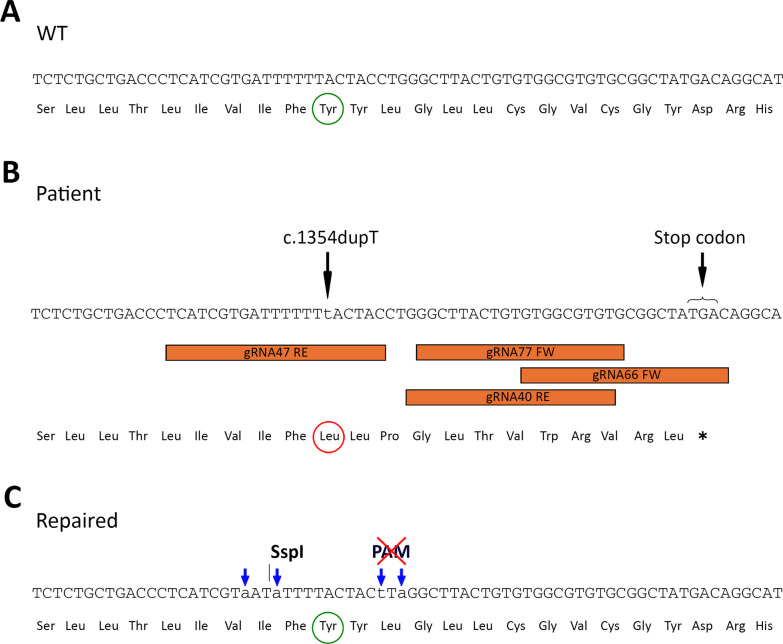
Design of a gene editing strategy for repairing the c.1354dupT (p.Tyr452 Leufs*13) mutation in the exon 13 of the *PROM1* gene. **A** In silico representation of the target sequence in a WT case. The amino acid affected by target mutation “Tyrosine” is highlighted in a green circle **B** In silico representation of a patient affect by the c.1354dupT (p.Tyr452 Leufs*13). The mutation is indicated by a black arrow and represented by a lowercase “t”. The Leucine generated due the mutation’s frameshift is highlighted in a red circle, and the consequent stop codon is indicated by a black arrow. The localization of the four designed *gRNAs (40RE, 47RE, 66FW and 77FW)* are framed in orange **C** In silico representation of the target sequence in a genetically repaired case. Blue arrows indicate silent mutations that introduce a restriction site for the Ssp1 and destroy the Protospacer Adjacent Motif (PAM) sequence to prevent the edited allele from being targeted again by the gRNA. The amino acid affected by target mutation “Tyrosine” is highlighted in a green circle. The WT reading frame is recovered

The repair oligonucleotides (Alt-R HDR Donor Oligos) were engineered to include silent mutations, near the target mutation site, using the web tool Silent Mutator (https://molbiotools.com/silentmutator.php) [54] and the DNAStar Lasergene software (v.7.1, DNAstar). Two of these silent mutations were introduce to create a new restriction site for the Ssp1 enzyme (Thermo Fisher, MA, USA, Cat# ER0771), allowing the recognition of proper integration of the repair oligonucleotide. The other two silent mutations were designed to destroy the Protospacer Adjacent Motif (PAM) sequence, preventing the edited allele from being targeted repeatedly by the gRNA (Fig. 2).

The predicted gene-editing efficiency of the designed four gRNA guides, were tested in vitro by editing the U2OS osteosarcoma cell line (ATCC HTB-96). The U2OS cells (1 × 10^6^ cells) were transfected with each gRNA (1 µg), the corresponding repair oligonucleotide (15µL at 10 µM), and the Cas9-protein v2 TrueCut™ (5 µg; Invitrogen, Paisley, UK. Cat# A36498) using the 100 µL pipette tip of an Neon Transfection System (Thermo Fisher, MA, USA. Cat# MPK5000), under the following pulse conditions: 1230 V; 10 Ms; 4 pulses. After 24 h of culture, gDNA from each essay was extracted, and the region containing the target mutation was amplified by conventional PCR using the *PROM1seq* primer. The PCR products were purified using the Wizard® PCR Preps DNA Purification System (Promega, Wisconsin, USA. Cat# A7170) and digested with the Ssp1(Thermo Fisher, MA, USA, Cat# ER0771) enzyme for 3 h at 37 °C to ascertain the most effective CRISPR/Cas9 editing. Untransfected U2OS cells served as the wild type (WT) control.

#### Detection of gene-edited iPSC clones

As a screening method to identify successfully genetically repaired iPSC clones, a specific primer incorporating the silent mutations present in the selected repair oligonucleotide were designed (*ALELO40RE* primer) (Supplementary file 3). Due to the similarity between a WT and a gene edited sequence, amplified by the *ALELO40RE* primer, a gradient conventional PCR assay was conducted to determine the optimal annealing temperature to identified successfully genetically repaired iPSC clones.

To test this method, U2OS cells were transfected, under the previously described conditions, with the selected gRNA and repair oligonucleotide. After 24 h of culture, gDNA was extracted and a gradient conventional PCR was performed.

#### Patient-derived iPSC gene editing

After 5 days of culture, the iPSC lines derived from patients IRD1 and 2, were dissociated using Accutase (Stem Cells Technologies, Cambridge, UK. Cat# 07920) and resuspended in mTeSR™ Plus medium supplemented with CloneR™2 (Stem Cells Technologies, Cambridge, UK. Cat# 100–0276, 100–0691). A total of 1 × 10^6^ cells were electroporated with the selected gRNA (1,5µL at 1µL/µg), the repair oligonucleotide (15 µL at 20 µM) and Cas9-protein v2 TrueCut™ (1,5µL at 5µL/µg, Thermo Fisher, MA, USA. Cat# A36498) using the Neon Transfection System (Thermo Fisher, MA, USA. Cat# MPK5000), with the following pulse conditions: 1200 V; 30 Ms; 1 pulse. The electroporated cells were plated at a density of 50 cells/cm^2^ on Matrigel coated (23 µg/cm2; Corning Life Sciences, NY, USA. Cat# 11,593,620) 6-well plates (Thermo Fisher Scientific, MA, USA. Cat# 11,337,694) using mTeSR™ Plus medium supplemented with CloneR™2 (Stem Cells Technologies, Cambridge, UK. Cat# 100–0276, 100–0691) for 24 h. The mTeSR™ Plus medium (Stem Cells Technologies, Cambridge, UK. Cat# 100–0276) was refreshed daily until the colonies reached a sufficient size for manual picking. iPSC clones were subsequently plated in 24-well plates (Thermo Fisher, MA, USA. Cat# 142,475), duplicated and cryopreserved.

The gDNA was extracted from 30 clones of each patient-derived iPSC lines and was amplified via conventional PCR using the *PROM1seq* primer (Supplementary file 3). The PCR products of the selected clones were purified with the Wizard® PCR Preps DNA Purification System (Promega, Wisconsin, USA. Cat# A7170) and digested with the Ssp1 enzyme (Thermo Fisher, MA, USA, Cat# ER0771) for 3 h at 37 °C. Proper integration of the repair oligonucleotide in the selected clones was then confirmed by Sanger sequencing using the *PROM1seqFW primer* (Supplementary file 3). The gene-edited clones were assessed for their ability to encode Prominin-1 through CD133 flow cytometry and western blotting.

#### Flow cytometry

The iPSC line derived from patient IRD1, its corresponding genetically repaired iPSC line, and the iPSC control line [FiPS] Ctrl1-Ep6F-5 were labelled with an anti-CD133 antibody conjugated to allophycocyanin (APC) (Supplementary file 2), following the manufacturer’s protocol. The flow cytometry assay was performed using the Gallios Flow Cytometer (Beckman Coulter, Indianapolis, USA) and the data were processed with Kaluza Analysis Software (Beckman Coulter, Indianapolis, USA). iPSC lines without staining served as negative controls for the assay.

#### Western blotting

Proteins obtained from the iPSC line derived from patient IRD2, its corresponding repaired iPSC line, and the control iPSC line [FiPS] Ctrl1-Ep6F-5 were extracted using RIPA buffer (25 mM Tris–HCl pH 7.4, 1 mM EDTA, 150 mM NaCl, 1% NP-40, 1% sodium deoxycholate, 0.1% SDS) with added protease inhibitors. Protein samples (20 µg each) were separated by SDS-PAGE technique and subsequently transferred to PVDF membranes. The proteins CD133 and β-actin (as a loading control) were immunodetected using specific antibodies (Supplementary file 2). Images were acquired using a GS-800 Calibrated Densitometer (Bio-Rad, Madrid, Spain) and analysed with Quantity One software (Bio-Rad, Madrid, Spain).

## Results

### Patient selection, ophthalmological examination and CES results

**Fig. 3:**
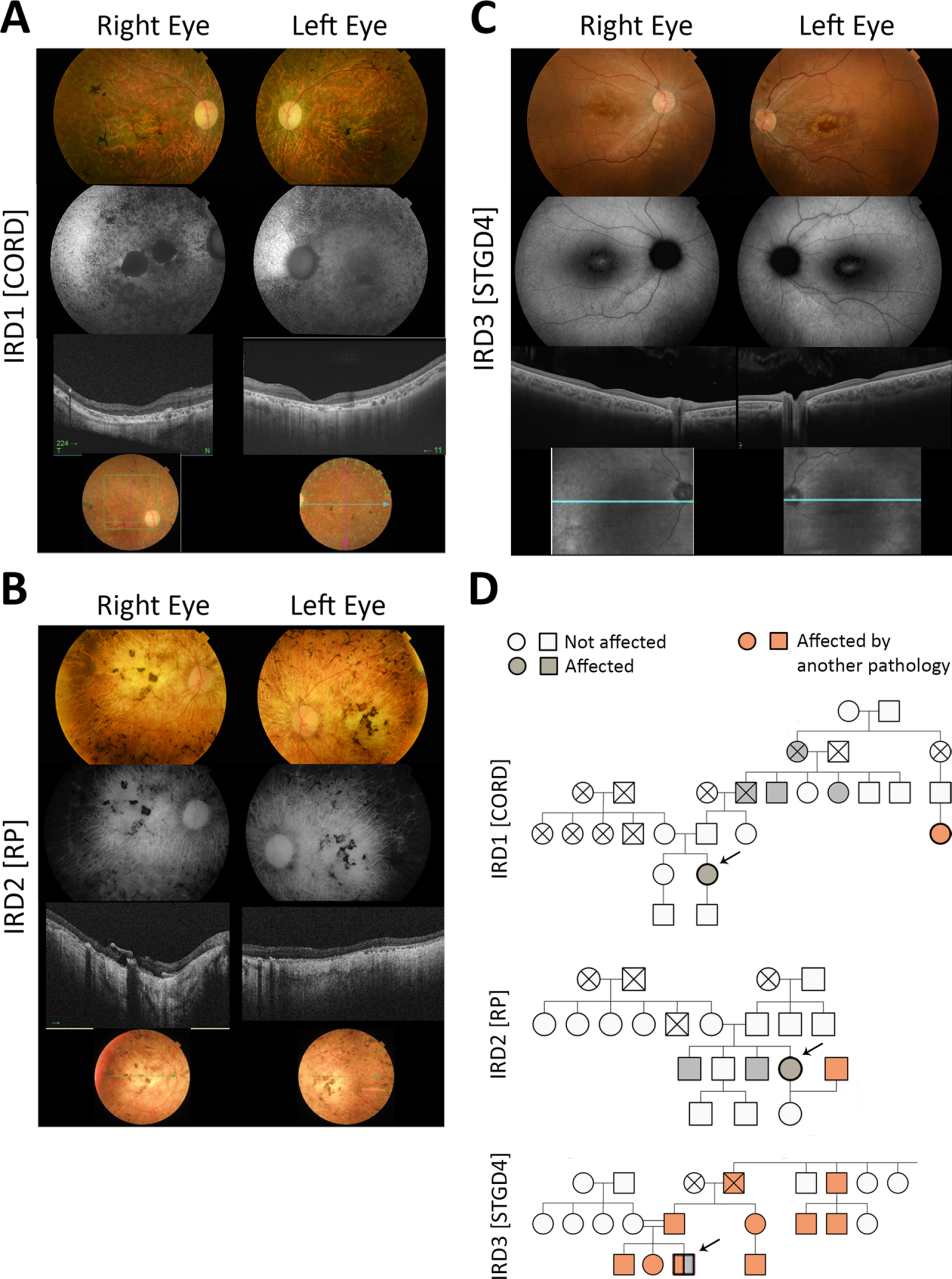
Phenotypic Variability of the c.1354dupT (p.Tr452 Leufs*13) Mutation in the *PROM1* Gene in Three Target Patients. **A** Patient IRD1 is a 47-year-old female displaying a CORD phenotype. Retinography reveals pigment in bone spicules, papillary pallor, macular atrophy, and vascular attenuation. Fundus Autofluorescence (FAF) displays patchy/mottled hypoautofluorescent patterns. Optical Coherence Tomotraphy (OCT) presents panretinal outer retinal layers (ORL) disruption with foveal involvement. **B** Patient IRD2 is a 54-year-old female exhibiting an RP phenotype. Retinography with bone spicules pigmentation, papillary pallor, macular atrophy, and vascular attenuation. FAF showing patchy/mottled hypoautofluorescent patterns. OCT reveals panretinal ORL disruption including the fovea. **C** Patient IRD3, a 20-year-old male, exhibits an STGD4 phenotype. Retinography displays Bull’s eye Maculopathy. FAF shows a hypoautofluorescent ring around the fovea. OCT exhibits ORL disruption of the previously described ring with preserved fovea, mainly affecting the RPE line but with photoreceptors still present in the macular area. **D** The pedigree analysis reveals an autosomal recessive (AR) inheritance pattern for all three target patients. IRD1’s paternal grandfather and two paternal great-uncles are affected; additionally, a cousin of his father received an RP diagnosis. That family resides in a small village in Cantabria and exhibits relatively high consanguinity. IRD2 has two affected male brothers and a non-affected son (her husband has congenital achromatopsia, highlighted in pink). IRD3 has no reported family history of ocular disease but few members present Charcot-Marie-Tooth (CMT) disease, highlighted in pink. The parents of IRD2 are second cousins, indicating consanguinity; IRD3’s father has CMT disease due to a duplication of the *PMP22* gene mutation in homozygosis, and IRD3 was heterozygous for this mutation as well, albeit asymptomatic

Eight patients with *PROM1*-related IRD were identified and studied at the IOBA’s clinical area. Their genetic background, clinical examination and functional and imaging characteristics are summarized in Tables 1, 2, and 3. Three of them presented retinopathy associated with the homozygous c.1354dupT (p.Tr452 Leufs*13) mutation in the *PROM1* gene inherited in an autosomal recessive manner, and were selected for the generation of iPSC lines, as they exhibited three distinct phenotypes: IRD1 is a 47-year-old female with the CORD phenotype (Fig. 3A); IRD2 is a 54-year-old female with the RP phenotype (Fig. 3B); and IRD3 is a 20-year-old male with the STGD4 phenotype (Fig. 3C). Relatives of the target patients included: a 46-year-old female, who is the sister of IRD1; a 21-year-old female, who is the daughter of IRD2; and a 52-year-old female, who is the mother of IRD3. None of these relatives exhibited abnormalities upon ophthalmological examination. The results of the CES are presented in Table 4.

**Table 2:** Clinical characteristics of the cohort of 8 patients with *PROM1*-related retinopathies

Patient	Phenotype	Age of onset (years)	Symptoms	Refractive error	BCVA LogMAR/age first visit	BCVA LogMAR/age second visit	BCVA LogMAR/age last visit	Family history	Associated pathology
IRD 1	CORD	6	Loss of AV followed by loss of VF	Hypermetropia	RE: 1.3/23 LE: 1.0	RE: 3.0/34 LE: 1.3	OD: 3.0/46 OD: 3.0	Paternal grandfather, two paternal great-uncles affected. A cousin of his father's affected by RP (great-grandparents with relative consanguinity)	Nistagmus, pseudophakia
IRD 2	RP	5	Diagnosed at the age of 5 upon revision following her brother's diagnosis. Simultaneous loss of VA and VF at 7 years of age, and nyctalopia at 19 years old. No visual rest at the age of 33	Hypermetropia	RE:1.0/14 LE: 1.0	RE: LP/45 LE: LP	OD: LP/54 OI: LP	2 affected male brothers	Nistagmus, strabismus, pseudophakia
IRD 3	STGD4	13	Loss of AV and photophobia	Mild myopia	RE: 0.4/17 LE: 0.5	RE: 0.3/18 LE: 0.4	OD: 0.5/20 OI: 0.6	No Ocular disease. Father Charcot-Marie-Tooth & patient asymptomatic have the *PMMD2* gene duplicated. Consanguinity (grandfathers were cousins)	No
IRD 4	CORD	3	Loss of AV followed by loss of VF, glare	Moderate myopia	NA	NA	OD: 3.0/41 OI: 3.0	1 affected male brother	Nistagmus
IRD 5	CORD	12	Loss of AV and photophobia, followed by dyschromatopsia, glare and loss of VF	High myopia	RE: 0.4/12 LE: 0.4	RE: 1.0/23 LE: 1.0	OD: 3.0/32 OI: 3.0	3 affected brothers	Nistagmus
IRD 6	STGD4	31	Loss of AV and metamorphopsia	Mild myopia, high astigmatism	RE: 0.3/31 LE: 0.2	RE: 0.10/32 LE: 0.10	OD: 0.00/39 OI: 0.92	Daughter possibly affected	No
IRD 7	STGD4	25	Loss of AV	Emmetropia	RE:0.1/30 LE: 0.17	RE:0.5/31 LE: 0.17	OD:0.1/37 OI: 0.3	1 affected male brother with more widespread disease. The father and one paternal uncle are also affected	Strabismus, cataract
IRD 8	CORD	13	Loss of AV and photophobia	Mild hypermetropia. astigmatism	RE: LP/40 LE: LP	RE: LP/48 LE: LP	RE: NLP/64 LE: NLP	2 cousins from the paternal family (male and female) affected RP. Relative consanguinity, as all are from a village with a population of fewer than 100 inhabitants	Nistagmus, cataract

IRD: Inherited retinal dystrophy. BCVA: best corrected visual acuity. VF: visual field. RE: right eye. LE: left eye. LP: light perception. NLP: no light perception. CORD: cone – Rod Dystrophy. NA: not available. STGD4: Stargardt’s disease type 4. RP: retinitis pigmentosa

**Table 3:** Additional functional and imaging characteristics of the identified patients with *PROM1*-related retinopathy

Patient	Fundoscopy	FAF Pattern			VF			ERG				OCT				
		Patched /mottled posterior pole HºAF	H^er^AF ring	Preserved fovea	Periphery HºAF	Central scotoma	Concentric retraction	Cone-rod pattern	Rod-Cone pattern	Subnormal scotopic and photopic	Abolished scotopic and photopic	ONL disruption in the macula with preserved fovea	ONL disruption in the macula without preserved fovea	Panretinal ONL disruption	SR deposits	H^er^refl foci
IRD 1	Macular atrophy, pigment in bone spicules, papillary pallor, vascular attenuation	Yes	–	–	Yes	Abolished	–	–	–	Yes	–	Yes	Yes	Yes	Yes	
IRD 2	Pigment in bone spicules, papillary pallor, macular atrophy, vascular attenuation	Yes	–	–	–	Abolished	–	–	–	Yes	–	Yes	Yes	Yes	–	
IRD 3	Bull’s eye maculopathy	–	Yes	–	–	Yes	–	Only subnormal photopic	Yes	–	–	Yes	Yes			
IRD 4	Macular atrophy, mild pigmentation in bone spicules & atrophic plaques in the inferior periphery	Yes	–	–	–	NA	NA	NA	NA	NA	Yes	Yes	–	–	Yes	Yes
IRD 5	Macular atrophy, pigment in bone spicules & atrophic plaques in the mid–periphery, papillary pallor, vascular attenuation	Yes	–	–	–	Abolished	–	–	–	Yes	–	Yes	Yes	Yes	–	
IRD 6	Macular atrophy without flecks	Yes	–	–	–	Yes	–	Only subnormal photopic	–	Yes	–	Yes	Yes			
IRD 7	Bull’s eye maculopathy, optic nerve pallor, macular atrophy, vascular attenuation, whitish mottling	Yes	Yes	–	Yes	Abolished	NA	NA	NA	NA	Yes	–	Yes	Yes	–	
IRD 8	Pigment in bone spicules and atrophic plaques in the periphery, macular atrophy, vascular attenuation	Yes	–	–	–	Abolished	–	–	–	Yes	–	Yes	Yes/ Choroid absent	Yes	Yes	

Abbreviations. FAF: Fundus autofluorescence. VF: visual field. ERG: Electroretinogram. OCT: optical coherence tomography. SR: subretinal. H^er^refl: hyperreflective. NA: not available. HºAF: hypoautofluorescence. H^er^AF: hyperautofluorescence. ONL: outer nuclear layer. IRD: Inherited retinal dystrophy

**Table 4:** Clinical exome sequencing results in patients with IRD related to the homozygous c.1354dupT mutation in the *PROM1* gene and their healthy relatives

Subject	Phenotype	Gene	RefSeq	Exon	Mutation	Classification	Genotype
IRD1	CORD	*PROM1*	NM_006017.3	13	c.1354dupT (p.Tr452 Leufs*13)	Pathogenic	Homozygosity
		*C2*	NM_000063.6	6	c.841_849 + 19del	Probably pathogenic [67]	Heterozygosity
IRD1’s sister	Control	*PROM1*	NM_006017.3	13	c.1354dupT (p.Tr452 Leufs*13)	Pathogenic	Heterozygosity
		*POMT1*	NM_001077365.2	20	c.2097C > A (p.Tyr699*)	Pathogenic	Heterozygosity
IRD2	RP	*PROM1*	NM_006017.3	13	c.1354dupT (p.Tr452 Leufs*13)	Pathogenic	Homozygosity
IRD2’s daughter	Control	*PROM1*	NM_006017.3	13	c.1354dupT (p.Tr452 Leufs*13)	Pathogenic	Heterozygosity
		*CNGB3*	NM_019098.5	10	c.1148del (p.Thr383fs)	Probably pathogenic	Heterozygosity
		*HYDIN*	NM_001270974.2	67	c.11461C > T (p.Arg3821*)	Probably pathogenic	Heterozygosity
IRD3	STGD4	*PROM1*	NM_006017.3	13	c.1354dupT (p.Tr452 Leufs*13)	Pathogenic	Homozygosity
		*SAMD11*	NM_001385641.1	14	c.2377C > T (p.Arg793*)	Probably pathogenic [65]	Heterozygosity
		*PEX6*	NM_000287.4	8	c.1802G > A (p.Arg601Gln)	Probably pathogenic [66]	Heterozygosity
IRD3’s mother	Control	*PROM1*	NM_006017.3	13	c.1354dupT (p.Tyr452fs)	Pathogenic	Heterozygosity
		*PEX6*	NM_000287.4	8	c.1802G > A (p.Arg601Gln)	Probably pathogenic	Heterozygosity

The sequencing data supporting the results reported in this table are suitable as supplementary material in Supplementary files 6, 7, 8, 9, 10, 11

### iPSC generation and characterization

*Generation of Patient’s iPSC Lines.* A primary fibroblast cell line was isolated from the dermal biopsy of each patient. The fibroblast lines: [CORD]-hFb, [RP]-hFb, and [STGD4]-hFb, were generated from the corresponding patient. The cell line populations showed the typical fibroblast-like morphology at phase contrast microscopy examination. Patient fibroblasts lines were reprogrammed into three iPSC lines, one for each phenotype. The generated iPSC lines: [CORD]-FiPSC1-Ep5F-2, [RP]-FiPSC1-Ep5F-10, [STGD4]-FiPSC1-Ep5F-8; displayed compact colonies with distinct borders, well-defined edges, and large nuclei.

**Fig. 4:**
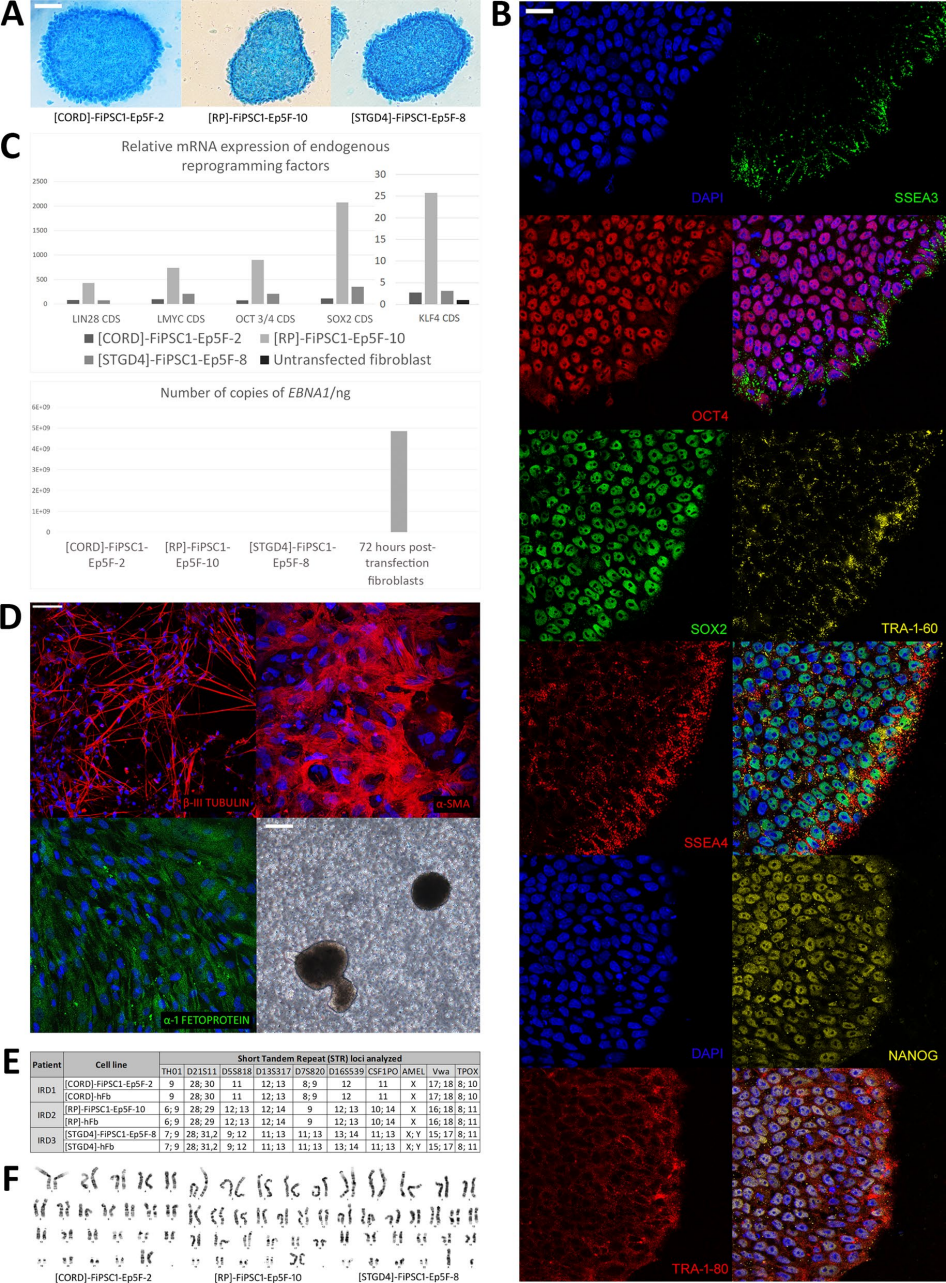
Characterization of the generated patient-derived iPSC lines. Alkaline phosphatase (AP) reactivity analysis.** A** Positive AP test results for all generated iPSC lines: [CORD]-FiPSC1-Ep5F-2, [RP]-FiPSC1-Ep5F-10, [STGD4]-FiPSC1-Ep5F-8. 50 µm scale. **B** Pluripotency analysis. Immunoreactivity of [RP]-FiPSC1-Ep5F-10 for pluripotency markers OCT4, SSEA3, SOX2, SSEA4, TRA1-60, NANOG, and TRA-1–81. 25 µm scale. **C** Molecular analysis of all generated iPSC lines. Relative mRNA expression of endogenous reprogramming factors (SOX2, OCT3/4, KLF4, LMYC, LIN28) apparently higher in the patient’s iPSC lines in comparison to unstransfected fibroblast. Above, the number of copies of the *EBNA1* gene evidently lower in the generated iPSC lines than in 72 h post-transfection fibroblast, electroporated in the same reprogramming conditions. **D** Analysis of the functional pluripotency of embryoid bodies (EB) derived from [RP]-FiPSC1-Ep5F-10. Immunoreactivity to ectoderm (β-III Tubulin), mesoderm (α-SMA) and endoderm (α-1 Fetoprotein) markers, along with phase contrast micrography of EB. 50 µm and 200 µm scale. **E** Identity analysis of the generated patient-derived iPSC lines. Correspondence between the patient’s fibroblast lines: [RP]-hFb, [STGD4]-hFb, [CRD]-hFb, and their respective patient-derived iPSC line for the Short Tandem Repetition (STR) loci: CSF1PO, D13S317, D16S539, D21S11, D5S818, D7S820, TH01, TPOX y vWA, and Amelogenin. **F** Cytogenetic integrity of the generated iPSC lines. G-banded metaphase analysis of the patient-derived iPSC lines

*AP staining and Pluripotency Immunocytochemistry*. The generated iPSC lines tested positive for AP activity, exhibiting distinct blue staining (Fig. 4A). Furthermore, all of them displayed immunoreactivity for the nuclear markers (OCT4, Nanog, Sox2) and cell membrane markers (SSEA-3, SSEA-4, Tra 1–60, Tra 1–80) associated with pluripotency (Fig. 4B; Supplementary file 4A).

*Molecular Analysis of the Reprogramming Factors*. The relative mRNA expression of the endogenous reprograming factors (*SOX2, OCT3/4, KLF4, LMYC, LIN28*) were apparently higher in the patient’s iPSC lines compared to the control. Besides, the number of copies of the *EBNA1* gene was lower in the generated iPSC lines than in the control. Furthermore, the relative mRNA expression of the exogenous reprogramming vectors was lower in the iPSC lines than in the control (data available on request) (Fig. 4C).

In Vitro* Differentiation*. The cell populations that emerged from the EB of each patient-derived iPSC line expressed immunoreactivity for specific markers of each germinal layer. The cytoplasm of cell populations induced to undergo endodermal differentiation were immunoreactive to α-1 Fetoprotein. Cell populations exposed to mesodermal differentiation showed a dense filamentous pattern of immunoreactivity to α- Smooth Muscle Actin (α-SMA). Meanwhile, cell populations treated with ectodermal differentiation medium expressed a thin filamentous pattern of β-III Tubulin immunoreactivity in their cytoplasm (Fig. 4D; Supplementary file 4B).

*DNA Fingerprinting, Karyotyping and Yarget Mutation Sequencing*. Allelic variants for each analysed STR locus showed correspondence between the patient’s fibroblast lines and their corresponding iPSC lines (Fig. 4E). In addition, cytogenetic analysis did not detect any structural chromosomal abnormality in the analysed metaphases of the patient’s iPSC lines (Fig. 4F). Finally, the presence of the target mutation c.1354dupT was confirmed by Sanger sequencing in all patient’s iPSC lines.

### Genetic repair of patient-derived iPSC lines

#### gRNA selection and detection of gene-edited iPSC clones

**Fig. 5:**
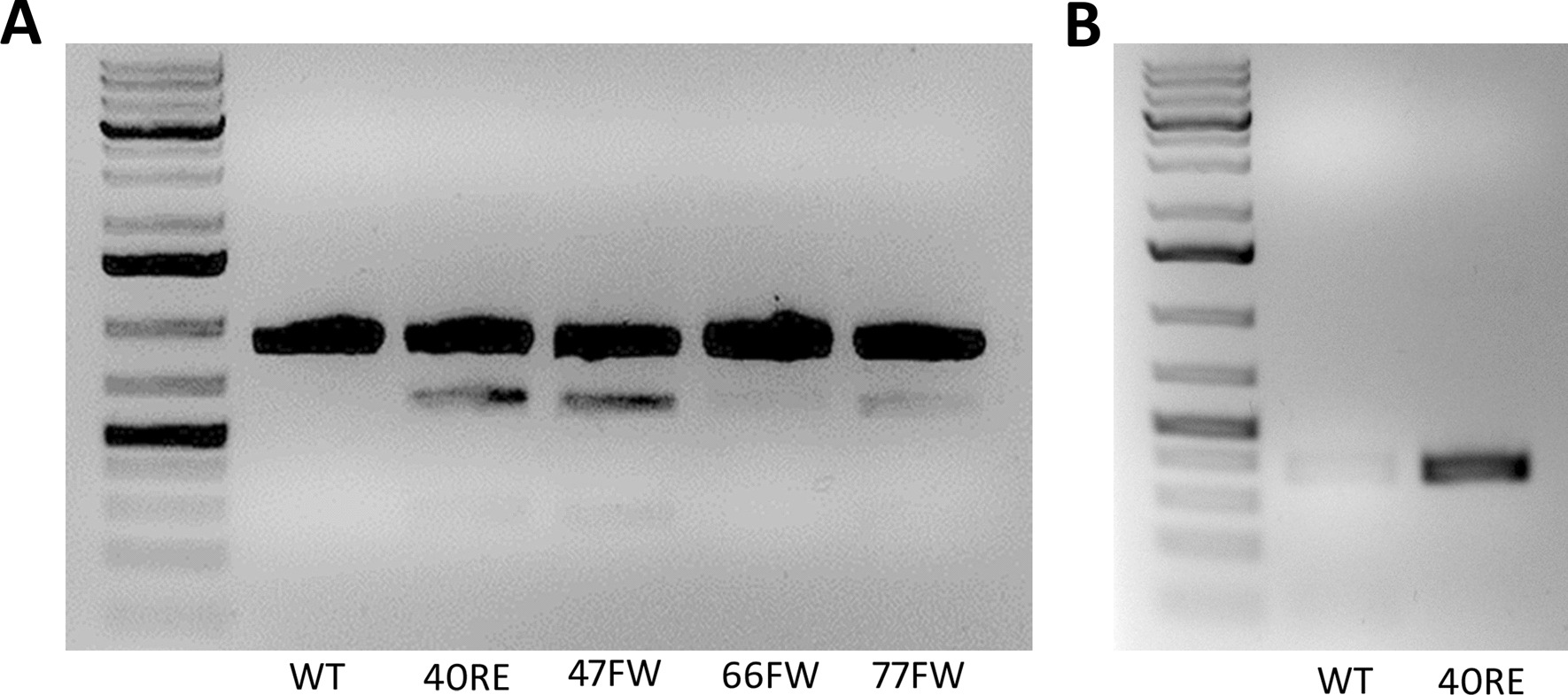
gRNA selection and detection of gene-edited iPSC clones (**A**) Comparison of gene editing efficiency among different *gRNAs (40RE, 47RE, 66FW, 77FW)*. Ssp1 digestion of PCR products obtained from gDNA of U2OS cells edited with each gRNA and its respective repair oligonucleotide. Untransfected U2OS cells served wild type (WT) control (**B**) Screening test for detection of genetically repaired clones. A comparison of conventional PCR products using the *ALELO40RE* primer between untransfected U2OS cells (WT) and U2OS cells transfected with *gRNA 40RE* and the corresponding *repair oligonucleotide 40RE* (40RE)

The *gRNA40RE, 47RE, 77FW* showed similar gene editing efficiency. The *gRNA40RE* (ACACGCCACACAGTAAGCCC) and the repair oligonucleotide *40RE* (TCCAGGTGGCTGGGTGGCCTGGTCATCTGCTCTCTGCTGACCCTCATCGTAATATTTTACTACTTGGGCTTACTGTGTGGCGTGTGCGGCTATGACAGGCATGCCACCCCGACCAC) were selected (Fig. 5A). Additionally, it was determined that using the *ALELO40RE* primer at 56 °C of annealing temperature, it was possible to differentiate between a WT sequence from a gene edited sequence (Fig. 5B).

**Fig. 6:**
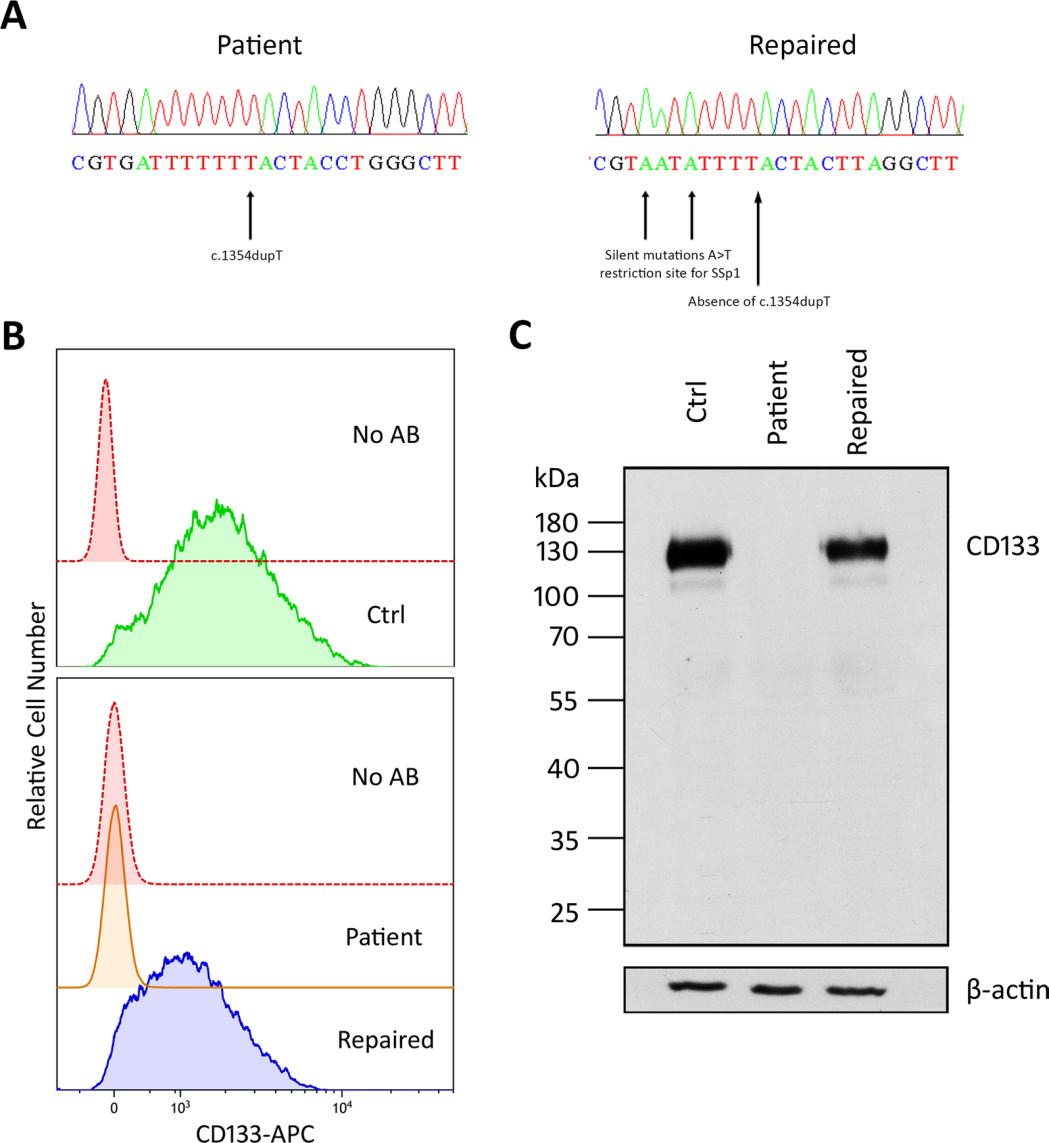
Genetic restoration in the gene-edited iPSC lines derived from patient IRD1 and IRD2. **A** Comparison between sequencing electropherograms of a patient-derived iPSC line and its corresponding genetically repaired iPSC line. In the patient’s sequence the target mutation c.1354dupT is pointed by an arrow. In the repaired sequence the silent mutations (A > T) that generates the restriction site for the Ssp1 enzyme are pointed by arrows. **B** Flow cytometry histograms illustrating the relative number of CD133-positive cells in the iPSC control line iPSC control line [FiPS] Ctrl1-Ep6F-5, the iPSC line derived from patient IRD1 [CORD]-FiPSC1-Ep5F-2, and its corresponding genetically repaired iPSC line [CORD]-FiPSC1-Ep5F-2-GC1. **C** Western blotting results depicting CD133 protein expression in the iPSC control line [FiPS] Ctrl1-Ep6F-5, the iPSC line derived from patient IRD2 [RP]-FiPSC1-Ep5F-10, and its corresponding genetically repaired iPSC line [RP]-FiPSC1-Ep5F-10-GC1. Full-length gels are available in the figure at the Supplementary file 5

The target mutation c.1354dupT (p.Tr452 Leufs*13) was corrected in the iPSC lines derived from patients IRD1 and 2, resulting in the generation of the gene-edited iPSC lines: [CORD]-FiPSC1-Ep5F-2-GC1 and [RP]-FiPSC1-Ep5F-10-GC1, respectively. Successful gene-edited was obtained in 10% of the clones selected from each patient. Proper integration of the repair oligonucleotide was confirmed by Sanger sequencing in both gene-edited iPSC lines (Fig. 6A).

### CD133 expression by the repaired iPSC lines

The quantification of the CD133-positive cell population in the flow cytometry assay revealed high CD133 expression in the iPSC control line [FiPS] Ctrl1-Ep6F-5, no expression in the iPSC line derived from patient IRD1, which was comparable to the non-stained negative control, and restoration of expression in the gene-edited iPSC line derived from patient IRD1 (Fig. 6B). In the western blotting assay, the CD133 was highly expressed in the iPSC control line, undetectable in the iPSC derived from patient IRD2, and its expression recovery was confirmed in the gene-edited iPSC line from patient IRD2 (Fig. 6C).

## Discussion

I this study, we report a cohort of eight *PROM1*-related IRD patients, including three individuals harbouring the same mutation (c.1354dupT) but expressing different phenotypes (CORD, RP and STG4). We selected these three patients to elucidate the pleiotropic effect of the c.1354dupT in the *PROM1* gene. To assess this matter in question, we expanded their genetic panel using CES and generated patient-derived iPSC to model the disease. Furthermore, we genetically repaired these iPSC as the first step towards designing therapeutic strategies. We plan to further study the disease by continuing our research in this direction.

Few cohort studies of *PROM1*-related retinopathies have been reported as it is a rare disease. In this regard, mutations in this gene account for 1 to 9.5% of AR CORD worldwide [8, 55, 56], 2% of AR RP and 4% AD RP in Spain [57]. In Asia, a cohort of 10 Japanese patients, considered as large cohort, were related to 3 variants, all exhibiting AD inheritance [58]; similarly, 10 Korean patients were all related to the same variant (p.Arg373Cys), also showing AD inheritance [25]. In Europe, the largest cohort included 25 Spanish patients, but only 7 of them underwent the main outcome measures FAF and OCT [22]; meanwhile, the second largest study included only 19 European patients [23]. In that sense, we report a non-negligible number of patients, including 5 different pathogenic *PROM1* variants, heterogeneous phenotypical characteristics, both sexes between 20 and 64 years old, exhibiting AD and AR inheritance patterns, and main ophthalmology imaging (FAF and OCT) was performed in all of them.

Our target mutation c.1354dupT (p.Tyr452Leufs*) is a complete loss-of-function variant (LOF) resulting in a premature stop codon. Similar to our study, in the two largest European cohort studies [22, 23], the c.1354dupT mutation was the most prevalent variant, highlighting its importance in the European population. Furthermore, its previously unreported pleiotropic effect justified focusing the experimental part of this study on these patients.

The c.1354dupT mutation has been associated with early onset AR phenotypes of severe panretinal dystrophy and late onset moderate MD [23]; and AR forms of CORD and RP [22]. Additionally, we report an early-medium onset (13 years old) of an AR form of STGD4 in patient IRD3 (aged 20 years old), contrary to the typical late onset AD manifestation of STGD4 dystrophy with an evident bull’s eye maculopathy [24, 59]. Nevertheless, given the young age of this patient we cannot rule out that he will be evolving to CORD later in his live. Interestingly, this patient has also a duplication of the *PMP22* gene, associated with Charcot-Marie-Tooth disease, although he remains asymptomatic.

Currently, it remains challenging to determinate whether *PROM1*-related dystrophies originate in the RPE or at the photoreceptors base due to its intricate role in the visual processes, participating in OS morphogenesis, RPE autophagy, and glial apoptosis [28–31]. At a clinical level, we could attempt to deduce it from the OCT and ERG results. We have observed in our series is that in older patients, and therefore with a more advanced condition, both RPE and photoreceptors are absent on OCT. However, interestingly, the two youngest patients, IRD 3 and IRD7, exhibit alteration of the RPE line with the photoreceptor line still present, as does IRD5 reviewed at 41 years of age, who presents a CORD phenotype and had the photoreceptor line preserved in the OCT. This leads us to suspect that at least in these patients, the disease primarily affects the RPE. However, IRD3 and IRD5 have photoreceptor function affected on ERG. Consequently, it would be impossible to definitively establish the primary affected cell type solely by observing these clinical features, since some IRDs affect the function of specific cell types without the disappearance of the corresponding retinal layer in histological studies or imaging tests until we are facing advanced stages of the disease.

Additionally, as supported by our study, most authors agree that early macular involvement is a common feature for *PROM1*-related IRD patients [60]. Furthermore, for *PROM1*-related STGD4 disease, it appears that pathological changes primarily affect the photoreceptors rather than the RPE. However, to deeply assess this matter, prospective studies conducting serial SD-OCT on a large cohort of *PROM1*-related IRD patients from the onset of symptoms and over several years would be necessary. This approach is outlined in the unique prospective cohort study of *PROM1*-associated retinal degeneration, examined with OCT but only for a 24-month duration [59].

Pleiotropic effects of IRD-related genes seem to be a common phenomenon in clinics, still poorly studied, and understood. The variant c.2539G > A, p.Glu847Lys in the *HK1* gene has been associated to AD forms of MD, CORD and RP [12, 13]. Similarly, the c.629C > G, p.Pro210Arg variant in the *PRPH2* gene with AD forms of macular dystrophy and RP [15, 16]. Additionally, similar to our target mutation, the c.1117C > T, p.Arg373Cys variant in the *PROM1* gene, has been associated with AD forms of STGD4 [25, 61, 62], CORD [23–25] and RP [22, 23], displaying phenotypical variability even within the same family. Although presently unexplained, this phenomenon is likely related to modifier genes affecting penetrance, dominance, expressivity, and pleiotropy of the IRD-causing variant [63].

The CES evaluation detected different associated monoallelic variants in two target patients that are recognised for being associated to AR phenotypes [64–67]; however, we cannot completely exclude nor confirm their phenotypic influence [68]. In IRD1, the monoallelic c.841_849 + 19del variant in the *C2* gene is associated with complement component 2 deficiency through skipping of exon 6 during RNA splicing [67]. Many *C2* variants has been associated with Age-related Macular Degeneration (AMD) [69]. However, the c.841_849 + 19del variant has not been associated with this pathology yet; nevertheless, due its early frameshift effect, it may cause similar microglia dysregulation as other AMD-related *C2* variants [70].

Besides, the monoallelic c.2377C > T (p.Arg793*) variant in the *SAMD11* gene found in IRD3 has been related to AR RP due to its interaction with the photoreceptor-specific transcription factor Cone-Rod homeobox (CRX) [65] and the Polycomb repressive complex 1 component (PRC1) [71], both implicated in the photoreceptor differentiation. Besides, the overexpression of the PCGF1 subunit of the PRC1 in cancer stem cells has been associated with an enhanced expression of CD133 [72]. Whereas, the monoallelic c.1802G > A (p.Arg601Gln) variant in the *PEX6* gene has been associated with Peroxisome biogenesis disorders [66]. Additionally, CD133 has been associated with upregulation of Peroxisome proliferator-activated receptor alpha in cancer stem cells, which is essential for retinal lipid metabolism [73, 74]. Thus, it is plausible to correlate CD133 deficiency with a possible monoallelic alteration in photoreceptors differentiation and retinal peroxisome function in the IRD3 patient.

To clarify the possible influence of the mentioned monoallelic variants in the *PROM1*-related IRD phenotypes, it would be convenient to generate gene-edited patient-derived iPSC and murine models to study the associated variants independently. However, at present, none of these variants could be stated as modifier genes as none of them participate directly in the described Prominin-1 metabolic or function pathways [27–31, 75, 76]. On the other hand, other variants not related to retinal disease, and therefore not included in this study, may have some influence in this phenomenon. Further studies should include more comprehensive molecular diagnosis methods, such as whole exome or genome sequencing.

The generated patient-derived iPSC lines: [CORD]-FiPSC1-Ep5F-2, [RP]-FiPSC1-Ep5F-10, [STGD4]-FiPSC1-Ep5F-8, meet the characterization requirements the Carlos III Health Institute, the Spanish competent authority for iPSC banking and registration, in accordance with the guidelines established by the International Stem Cell Banking Initiative (ISCBI) [77]. In this sense, these iPSC lines can be considered as fully reprogrammed and functional iPSC. Although CD133 has been historically recognized as a stem cell marker, highly expressed in the cell membranes of undifferentiated cells such as ESC and iPSC [75, 78], our findings indicate that CD133 deficiency does not appear to affect reprogramming, pluripotency, or in vitro differentiation of iPSC. Furthermore, in contrast to CD133-KO ESC [76], cell proliferation, in terms of the number of passing days, did not appear to be affected when compared to the iPSC control line [FiPS] Ctrl1-Ep6F-5.

Although, *PROM1* has been previously knocked out in RPE cells [30, 78] and murine models [79–81], the application of CRISPR/Cas9 genome editing on patient-derived iPSC to establish IRD-specific disease models enables researchers to study the distinct molecular effects associated with each IRD-related variant, extending beyond the mere absence of a protein as in KO murine models [82, 83]. In this regard, the next steps in our research horizon include obtaining gene-edited iPSC-derived RPE cells to evaluate this matter and to future cell therapy assays as they seem to be the most promising alternatives for replacement therapies [84].

To the best of our knowledge, this is the first report of a *PROM1*-related IRD mutation genetically repaired in patient-derived iPSC lines. After confirming the functional restoration of the generated genetic-repaired iPSC, they can be useful tools for preclinical cell therapy studies as an alternative treatment to replace degenerated retina in advanced diseases. Furthermore, the gene editing strategy could contribute to the design of future gene therapy studies to treat early stages of *PROM1*-related retinal dystrophy. Finally, we would like to highlight the relevance of having a better understanding of the pleiotropism phenomena of IRD-related genes and variants, as it may be a crucial milestone in the proper diagnosis, classification, and treatment of IRD.

Limitations of the study include the extensive phenotypic and genotypic heterogeneity present within IRD, which complicates the identification of correlations between associated variants and the patient phenotypic traits, even when employing massive NGS techniques such as CES. Future studies should consider expanding the patient cohort, encompassing a broader spectrum of mutations, and incorporating whole exome/genome sequencing to achieve a more comprehensive understanding. Furthermore, deeper characterization of a gene-edited iPSC derivatives, including RPE, photoreceptors, and retinal organoids, are essential to elucidate *PROM1*-related IRD phenotypic variability and disease mechanisms, and finally to explore the potential therapeutic utility of gene editing or cell therapy product development.

## Conclusion

The c.1354dupT mutation in the *PROM1* gene is associated to three distinct AR phenotypes of IRD. This pleotropic effect might be related to the influence of monoallelic variants in other genes associated with retinal dystrophies, but further evidence needs to be provided. Future experiments should include gene-edited patient-derived iPSC due to its potential as disease modelling tools to elucidate this matter in question. The generated patient-derived iPSC lines are fully reprogrammed and functional. Besides, the gene-editing strategy successfully restored the capability of the *PROM1* gene to encode Prominin-1. After functional characterization, the gene-edited iPSC lines can be used as a basis for future development of gene and cell therapies for *PROM1*-related IRD patients.

### Supplementary Information

Supplementary file 1: List of the 998 genes related to retinal diseases evaluated in the clinical exome sequencing analysis.

Supplementary file 2: Antibodies and their experimental conditions.

Supplementary file 3: Primer sequences for iPSC molecular characterization, sequencing, and gene editing.

Supplementary file 4: Pluripotency analysis. (A) Immunoreactivity of [CORD]-FiPSC1-Ep5F-2 and [STGD4]-FiPSC1-Ep5F-8 for the pluripotency markers OCT4, SSEA3, SOX2, SSEA4, TRA1-60, NANOG, and TRA-1-81. 25 µm scale. (B) Analysis of the functional pluripotency of the embryoid bodies (EBs) derived from [CORD]-FiPSC1-Ep5F-2 and [STGD4]-FiPSC1-Ep5F-8. Immunoreactivity to ectoderm (β-III Tubulin), mesoderm (α-SMA) and endoderm (α-1 Fetoprotein) markers. 25 µm scale.

Supplementary file 5: Full-length gels of Figure 4. (A) Full-length gels of Western blotting of CD133 protein expression in the iPSC control line [FiPS] Ctrl1-Ep6F-5, the iPSC line derived from patient IRD2 [RP]-FiPSC1-Ep5F-10, and its corresponding genetically repaired iPSC line [RP]-FiPSC1-Ep5F-10-GC1. (B). Full-length gels of Western blotting of β-actin (control).

Supplementary file 6: Sequencing data from IRD1.

Supplementary file 7: Sequencing data from IRD1’ sister.

Supplementary file 8: Sequencing data from IRD2.

Supplementary file 9: Sequencing data from IRD2’ daughter.

Supplementary file 10: Sequencing data from IRD3.

Supplementary file 11: Sequencing data from IRD3’ mother.

## Data Availability

All data generated or analysed during this study are included within the article.

## Abbreviations

IRD: inherited retinal dystrophy
iPSC: induced pluripotent stem cell
RP: retinitis pigmentosa
PCD: progressive cone dystrophy
CORD: cone-rod dystrophy
MD: macular dystrophy
AD: autosomal dominant
AR: autosomal recessive
XL: X-linked
NGS: next generation sequencing
ES: exome sequencing
STGD4: stargardt’s disease type 4
OS: photoreceptor’s outer segments
ESC: embryonic stem cells
IOBA: institute for applied ophthalmobiology
IBGM: institute of molecular biology and genetics
CES: clinical exome sequencing
BCVA: best corrected visual acuity
FAF: fundus autofluorescence
VF: visual field
ERG: electroretinography
OCT: optical coherence tomography
AMD: age-related macular disease
FBS: fetal bovine serum
IMDM: Iscove′s modified Dulbecco′s medium
EDTA: ethylenediamine tetraacetic acid
AP: alkaline phosphatase
PBS: phosphatase-buffered saline
TBS: Tris-buffered saline
DAPI: 4',6-diamidino-2-fenilindol
cDNA: complementary DNA
mRNA: messenger RNA
qRT-PCR: quantitative reverse transcription PCR
STR: short tandem repeat
α-SMA: α- Smooth Muscle Actin
