# Supplementary material for: Clinical exome analysis and targeted gene repair of the c.1354dupT variant in iPSC lines from patients with PROM1-related retinopathies exhibiting diverse phenotypes
Source: Stem Cell Res Ther. 2024 Jul 2;15:192. doi: 10.1186/s13287-024-03804-2 (PMC11218195; doi:10.1186/s13287-024-03804-2)
Supplement: Supplementary file 1 — List of the 998 genes related to retinal diseases evaluated in the clinical exome sequencing analysis. [file 13287_2024_3804_MOESM1_ESM.docx]

**Supplementary material 1**.

*ADIPOR1, ARL6, BBIP1, BBS1, BBS2, BBS4, BBS5, BBS7, BBS9, BBS10, BBS12, C8orf37, CEP19, CEP290, IFT172, IFT27, INPP5E, KCNJ13, LZTFL1, MKKS, MKS1, NPHP1, SDCCAG8, TRIM32, TTC8, PRDM13, RGR, TEAD1, AIPL1, CRX, GUCA1A, GUCY2D, PITPNM3, PROM1, PRPH2, RIMS1, SEMA4A, UNC119, ABCA4, ADAM9, ATF6, C21orf2, CACNA2D4, CDHR1, CEP78, CERKL, CNGA3, CNGB3, CNNM4, WDR34, GNAT2, IFT81, KCNV2, PDE6C, PDE6H, POC1B, RAB28, RAX2, RDH5, RPGRIP1, SLC4A7, TTLL5, CACNA1F, RPGR, GNAT1, PDE6B, RHO, CABP4, GNB3, GPR179, GRK1, GRM6, LRIT3, SAG, SLC24A1, TRPM1, NYX, ESPN, WFS1, CDH23, CIB2, MYO7A, PCDH15, PDZD7, USH1C, IMPDH1, OTX2, CCT2, CLUAP1, CRB1, DTHD1, GDF6, IFT140, IQCB1, LCA5, LRAT, NMNAT1, RD3, RDH12, RPE65, SPATA7, TULP1, BEST1, C1QTNF5, CTNNA1, EFEMP1, ELOVL4, FSCN2, GUCA1B, HMCN1, IMPG1, RP1L1, TIMP3, CFH, DRAM2, MFSD8, VCAN, AFG3L2, MFN2, MIEF1, NR2F1, OPA1, ACO2, NBAS, RTN4IP1, TMEM126A, TIMM8A, ARL3, CA4, HK1, KIF3B, KLHL7, NR2E3, NRL, PRPF3, PRPF4, PRPF6, PRPF8, PRPF31, ROM1, RP1, RP9, SNRNP200, SPP2, TOPORS, AGBL5, ARHGEF18, AHR, ARL2BP, CLCC1, CLRN1, CNGA1, CNGB1, CWC27, CYP4V2, DHDDS, DHX38, EMC1, ENSA, EYS, FAM161A, GPR125, HGSNAT, IDH3B, IMPG2, KIAA1549, MAK, MERTK, MVK, NEK2, NEUROD1, c2orf71, PDE6A, PDE6G, POMGNT1, PRCD, PROS1, RBP3, REEP6, RLBP1, SAMD11, SLC7A14, TRNT1, USH2A, ZNF408, ZNF513, OFD1, RP2, ABCC6, ATXN7, COL11A1, COL2A1, JAG1, KIF11, OPA3, PAX2, TREX1, ABHD12, ACBD5, ADAMTS18, AHI1, ALMS1, CC2D2A, CEP164, CLN3, COL9A1, CSPP1, EXOSC2, FLVCR1, GNPTG, HARS, HMX1, INVS, LAMA1, LRP5, MTTP, NPHP3, NPHP4, PANK2, PCYT1A, PEX1, PEX2, PEX7, PHYH, PLK4, PNPLA6, POC5, PPT1, PRPS1, RDH11, RPGRIP1L, SLC25A46, TMEM216, TMEM237, TTPA, TUB, TUBGCP4, TUBGCP6, WDPCP, WDR19, ZNF423, GPR98, ARSG, CEP250, USH1G, CAPN5, ELOVL1, FZD4, ITM2B, MAPKAPK3, MIR204, OPN1SW, RB1, RCBTB1, TSPAN12, ASRGL1, C12orf65, CDH3, DYNC2H1, MFRP, OAT, PLA2G5, RBP4, RGS9, RGS9BP, MT-ATP6, MT-TH, MT-TL1, MT-TP, MT-TS2, CHM, DMD, NDP, OPN1LW, OPN1MW, PGK1, RS1, AARS, ABCC8, ACD, ACOX1, ACTA2, ACTB, ACTG1, ACTL6B, ACVRL1, ADAMTSL1, ADAMTSL4, ADAR, AGXT, AIRE, AKT1, ALDH1A3, ALDH3A2, ALDH6A1, ALG12, ALG6, ALG8, ALPK1, AMACR, ANK1, ANO10, AP3B2, AP3D1, AP5Z1, APC, APOB, APOC2, APOE, APPL1, ARL13B, ARL2, ARL6IP6, ARMC9, ARV1, ARVCF, ASAH1, ASPA, ASXL1, ATCAY, ATG7, ATOH7, ATP1A2, ATP1A3, ATP2B2, ATP6V0A2, ATP6V1A, ATP6V1E1, ATXN2, B3GALNT2, B3GNT1, B9D1, B9D2, BAP1, BAZ1B, BCL11A, BCL7B, BCOR, BCS1L, BLK, BLM, BLOC1S3, BLOC1S5, BMP4, WBSCR22, C12orf57, C2CD3, C4A, C9, CA2, CACNA1A, CACNA1B, CACNA2D1, CARS, CASK, CAV1, CBS, CCDC103, CCDC22, CCDC28B, CCDC39, CCDC40, CCDC65, CCM2, CCND1, CCNO, FAM58A, CCR1, CDK19, CDK4, CDKN2A, CDKN2B, CEL, CELF2, CENPF, CEP120, CEP41, PCDP1, C21ORF59, C11ORF70, CFHR1, CFHR3, CFI, CHD7, CHEK2, CHN1, CHRDL1, CHST14, CHST6, CLCN2, CLCN3, CLCNKB, CLDN19, CLEC3B, CLIP2, CLN5, CLN6, CLTC, CNKSR2, APOPT1, COG1, COL11A2, COL18A1, COL4A1, COL8A2, COL9A2, COL9A3, COMT, COQ2, COX15, COX7B, COX8A, CP, C5ORF42, CPLX1, CREBBP, ISPD, CRYAB, CST3, CTBP1, CTC1, CTLA4, CTNNB1, CTNS, CTSA, CTSD, CYFIP2, CYP1B1, CYP27A1, CYSLTR2, DACT1, DAG1, DALRD3, DARS, DCT, DDR2, DEAF1, DGCR2, DGCR6, DGCR8, DHX16, DLAT, DLK1, DLST, DNAAF1, LRRC6, DNAAF2, DNAAF3, DYX1C1, HEATR2, PIH1D3, DNAH1, DNAH11, DNAH5, DNAH9, DNAI1, DNAI2, DNAJB13, DNAJC21, DNAJC30, DNAL1, DNM1, DNM2, DNMT3A, DNMT3B, DPAGT1, DPM1, DPP6, DPYD, DRC1, DSE, DST, DUX4, WDR60, DYNC2LI1, DYRK1A, ECHS1, EDN3, EDNRB, EEF1A2, EIF4H, ELMO2, ELN, ENG, ENPP1, EPAS1, EPG5, ERAP1, ERCC1, ERCC2, ERCC3, ERCC4, ERCC5, ERCC6, ERCC8, DGCR14, ETHE1, EXOSC3, F12, FAM111A, FAS, FBLN1, FBLN5, FBN1, FBN2, FDXR, FGF12, FGF13, FGF3, FGFR1, FGFR2, FH, FIBP, FKBP6, FKRP, FKTN, FLCN, FLII, FLNB, FOXC1, FOXE3, FOXJ1, FOXRED1, FRG1, FZR1, G6PC, GABBR2, GABRA2, GABRA5, GABRB2, GABRG2, GALC, GALNT3, GAS2L2, GAS8, GATA3, GBA, GCDH, GCK, GDF2, GDF3, GGCX, GLB1, GM2A, GMPPB, GNA11, GNAQ, GNB5, GNPTAB, GP1BB, GPIHBP1, GPR143, GRHL2, GRIN2D, GRN, GSS, GTF2E2, GTF2H5, GTF2I, GTF2IRD1, GTF2IRD2, GZF1, HACE1, HADH, HADHA, HADHB, HBB, HBG1, HBG2, HCCS, HCN1, HEXA, HEXB, HHAT, HID1, HIRA, HKDC1, HLA-A, HLA-B, HLA-DPA1, HLA-DPB1, HLA-DRB1, HNF1A, HNF4A, HPS4, HPS5, HPS6, HSD11B2, HSD17B10, HSPD1, HYDIN, IDH3A, IDS, IDUA, IFNG, IFNGR1, IFT122, IFT52, IFT74, IFT80, IFT88, IGFBP7, IKBKG, IL10, IL12A, IL12A-AS1, IL12B, IL23R, INS, INSR, INTS1, IPO8, IQSEC2, ISCA1, IVNS1ABP, JMJD1C, KATNB1, KCNA2, KCNB1, KCNJ11, KIAA0586, KIF1B, KIF5A, KLF1, KLF11, KLRC4, KRAS, KRIT1, KRT25, KRT71, KRT74, LAMB2, LARGE, LETM1, LIG3, LIMK1, LIPH, LIPT1, LMNA, LOXL1, LOXL3, LPAR6, LPL, LRP2, LRRC32, LRRC56, LTBP2, LYST, MAB21L1, MAF, MAFB, MAGEL2, MAN2B1, MAN2C1, MAPRE2, MAX, MC1R, MCIDAS, MCOLN1, MDH2, MECR, MED12, MEFV, MEG3, WBSCR27, MGMT, MIA3, C19ORF70, MITF, MLX, MLXIPL, MMACHC, MORC2, MPDZ, MPLKIP, MSRB3, MSTO1, MT-ATP8, MT-CO1, MT-CO2, MT-CO3, MT-CYB, MT-ND1, MT-ND2, MT-ND3, MT-ND4, MT-ND4L, MT-ND5, MT-ND6, MT-TE, MT-TF, MT-TK, MT-TN, MT-TQ, MT-TS1, MT-TV, MT-TW, MTFMT, MTSS1L, MYD88, MYO5A, MYO6, MYOC, NAA10, NCAPG2, NCF1, NDE1, NDUFA10, NDUFA12, NDUFA13, NDUFA2, NDUFA4, NDUFA9, NDUFAF1, NDUFAF2, NDUFAF3, NDUFAF5, NDUFAF6, NDUFB11, NDUFB8, NDUFS1, NDUFS2, NDUFS3, NDUFS4, NDUFS7, NDUFS8, NDUFV1, NDUFV2, NECAP1, NEK1, NEK10, NEK8, NELFA, NEU1, NF1, NF2, NGLY1, NHS, NLRP3, NME8, NOD2, NOTCH2, NRCAM, WHSC1, NSMCE2, NTRK2, NUS1, OCA2, OCRL, CCDC114, ARMC4, CCDC151, TTC25, OVOL2, LEPREL1, PAK2, PARS2, PAX4, PAX6, PCNA, PDCD10, PDE6D, PDHA1, PDX1, PEPD, PET100, PEX10, PEX11B, PEX12, PEX13, PEX14, PEX16, PEX19, PEX26, PEX3, PEX5, PEX6, PGAP1, PIEZO2, PIGG, PIGL, PIK3CA, PISD, PLOD1, PMM2, POGZ, POLG, POLR3A, POMGNT2, POMK, POMT1, POMT2, PORCN, POT1, POU3F4, PPP2R3C, PPP3CA, PRDM5, PRDX1, PRF1, PRR12, PRSS56, PRTN3, PRUNE, PSAP, PTCH1, PTEN, PTPN22, PUF60, RAB18, RAB3GAP1, RAB3GAP2, RAI1, RAX, RELN, RERE, RET, RFC2, RIMS2, RMRP, RNASEH1, RNF113A, RNF216, RNU4ATAC, RNU7-1, RPL10, RPS6KA3, RREB1, RRM2B, RSPH1, RSPH3, RSPH4A, RSPH9, RTL1, RTTN, TMEM5, SACS, SALL1, SALL2, SALL4, SAR1B, SBDS, SCAPER, SCN3A, SCN8A, SCO2, SDHA, SDHAF1, SDHAF2, SDHB, SDHC, SDHD, SEC24C, EPT1, SEMA3E, SERPINC1, SF3B1, SH2B1, SH3BP2, SHH, SIM1, SIX3, SIX6, SLC12A6, SLC13A5, SLC19A2, SLC19A3, SLC1A2, SLC24A5, SLC25A11, SLC25A15, SLC35A2, SLC37A4, SLC38A3, SLC38A8, SLC45A2, SLC6A6, SMAD4, SMCHD1, SMPD1, SOX10, SOX2, SPAG1, SPEF2, SPG11, SPTBN1, SRD5A3, STAT3, STAT4, STK36, OBFC1, STUB1, STX1A, SUMF1, SURF1, SYCE1, SYNGAP1, SYNJ1, SZT2, TACO1, TAF2, TARS, TBC1D20, TBL2, TBX1, TBX22, TCTN1, TCTN2, TCTN3, TEK, TELO2, TENM3, TERC, TERF2IP, TERT, TFAP2A, TINF2, TK2, FAM57B, TLR4, TMCO1, TMEM107, TMEM127, TMEM138, TMEM218, TMEM231, WBSCR28, TMEM67, TMEM98, TNFRSF11A, TNFRSF11B, TP53, TPP1, TRAF3IP1, TRAK1, TRAPPC2, TRAPPC9, TRIM37, TRIM44, TRIP13, TSC1, TSC2, TTC12, TTC21B, TUBB, TUBB4B, C10ORF2, TXN2, TXNDC15, TYR, UBA5, UBAC2, UBE3B, UFD1L, UGP2, USP45, VHL, VPS13B, VPS33A, C16ORF62, VPS37D, VPS41, VPS4A, VSX1, WAC, WARS2, KIAA0196, WRN, WT1, WWOX, XRCC4, XYLT1, XYLT2, YAP1, YARS, YWHAG, ZEB1, ZEB2, ZFYVE26, ZMYND10, ZNF469, ZNF259, ARMS2, HTRA1, CFB, C2, C3, COL8A1, ABCA1, CETP, LIPC, ARHGAP21, B3GALTL, SKIV2L, CD46, CR1, CR2, CCDC41.*
