# Supplementary material for: Clinical exome analysis and targeted gene repair of the c.1354dupT variant in iPSC lines from patients with PROM1-related retinopathies exhibiting diverse phenotypes
Source: Stem Cell Res Ther. 2024 Jul 2;15:192. doi: 10.1186/s13287-024-03804-2 (PMC11218195; doi:10.1186/s13287-024-03804-2)
Supplement: Supplementary file 2 — Antibodies and their experimental conditions. [file 13287_2024_3804_MOESM2_ESM.docx]

**Additional file 2.** Antibodies and their experimental conditions

| **Primary antibody** | **Working solution** | **Incubation conditions** | **Secondary antibody** | **Working solution** | **Incubation conditions** |
| --- | --- | --- | --- | --- | --- |
| **Immunocytochemical analysis** | | | | | |
| Mouse IgG anti-OCT4 monoclonal antibody (Santa Cruz Cat# SC-5279, AB_628051) | 1:60 | 24 h 4ºC | Alexa 555 Donkey anti-mouse IgG (Thermo-Fisher Cat# A-31570, AB_2536180) | 1:200 | 2 h 37ºC |
| Rat IgM anti-SSEA-3 monoclonal antibody (Hybridoma Bank Cat # MC-631, AB_528476) | 1:50 | 24 h 4ºC | Alexa 488 goat anti-Rat IgM (Thermo Fisher Cat# A-21212, AB_2535798) | 1:200 | 2 h 37ºC |
| Rabbit IgG anti-SOX2 polyclonal antibody (Thermo Fisher Scientific Cat# PA1-16968, AB_2195781) | 1:100 | 24 h 4ºC | Alexa 488 Donkey anti-rabbit IgG (Thermo Fisher Cat# A-31572, AB_162543) | 1:200 | 2 h 37ºC |
| Mouse IgG anti-SSEA-4 monoclonal antibody (Hybridoma bank Cat# MC-813-70, AB_528477) | 1:50 | 24 h 4ºC | Alexa 555 Donkey anti-mouse igG (Thermo Fisher Cat# A-31570, AB_2536180) | 1:200 | 2 h 37ºC |
| Mouse IgM anti-TRA-1-60 monoclonal antibody (Millipore Cat# MAB4360, AB_2119183) | 1:200 | 24 h 4ºC | Alexa 647 Goat anti-mouse IgM (Thermo Fisher Cat# A-21238, AB_2535807) | 1:200 | 2 h 37ºC |
| Goat IgG anti-NANOG polyclonal antibody (R&D Cat# AF1997, AB_355097) | 1:50 | 24 h 4ºC | Alexa 647 Donkey anti-Goat IgG (Thermo Fisher Cat# A-21447, AB_2535864) | 1:200 | 2 h 37ºC |
| Mouse IgM anti-TRA-1-81 monoclonal antibody (Millipore Cat# MAB4381, AB_177638) | 1:200 | 24 h 4ºC | Cy3 Donkey anti-mouse IgM (Jackson Cat# 715-165-140, AB_2340812) | 1:200 | 2 h 37ºC |
| Mouse IgG2a anti-β-III Tubulin monoclonal AA10 antibody (Invitrogen Cat #480011, AB_2532242) | 1:400 | 24 h 4ºC | Alexa 555 Donkey anti-mouse IgG (Thermo-Fisher Cat# A-31570, AB_2536180) | 1:200 | 2 h 37ºC |
| Rabbit IgG anti-α-1 Fetoprotein Polyclonal antibody (Invitrogen Cat# PA5-21004, AB_11157055) | 1:400 | 24 h 4ºC | Alexa 488 Donkey anti-rabbit IgG (Thermo Fisher Cat# A-31572, AB_162543) | 1:200 | 2 h 37ºC |
| Mouse IgG1 anti-α-Smooth muscle actin 4A4 monoclonal antibody (Invitrogen Cat# MA5-15871, AB_11151920) | 1:400 | 24 h 4ºC | Alexa 555 Donkey anti-mouse IgG (Thermo Fisher Cat# A-31570, AB_2536180) | 1:200 | 2 h 37ºC |
| **Flow cytometry** |  |  |  |  |  |
| Mouse IgG1, kappa anti-CD133 monoclonal TMP4, APC (Thermo Fisher Cat# 17-1338-42, AB_1603199) | 5 µL (0.125 µg)/test | 1 h RT | - | - | - |
| **Western blotting** |  |  |  |  |  |
| Mouse IgG1 anti-CD133 monoclonal 2F8C5 antibody (Invitrogen Cat# MA1-219, AB_2725113) | 1:1000 | 24 h 4ºC | Mouse IgG, HRP-linked F(ab')₂ fragment from sheep (Amersham ECL, NA9310V) | 1:2000 | 2 h RT |
| Mouse IgG anti-β-actin-peroxidase monoclonal AC-15 antibody (Sigma-Aldrich Cat#A3854; AB_262011) | 1:1000 | 24 h 4ºC | - | - | - |

Abbreviation. RT: Room temperature.
