# Supplementary material for: Clinical exome analysis and targeted gene repair of the c.1354dupT variant in iPSC lines from patients with PROM1-related retinopathies exhibiting diverse phenotypes
Source: Stem Cell Res Ther. 2024 Jul 2;15:192. doi: 10.1186/s13287-024-03804-2 (PMC11218195; doi:10.1186/s13287-024-03804-2)
Supplement: Supplementary file 3 — Primer sequences for iPSC molecular characterization, sequencing, and gene editing. [file 13287_2024_3804_MOESM3_ESM.docx]

**Additional file 3.** Primer sequences for iPSC molecular characterization, sequencing, and gene editing

| **Name** | **Forward primer (5' – 3')** | **Reverse primer (5' – 3')** |
| --- | --- | --- |
| **iPSC molecular characterization** | | |
| *EBNA1copynumber* | GTCAAGGAGGTTCCAACCCG | TGGAAACCAGGGAGGCAAAT |
| *SOX2plasmid* | TTCACATGTCCCAGCACTAC | TTGTTTGACAGGAGCGACAA |
| *OCT3/4 plasmid* | AGGGCGAATTCACTCCTCAG | GTATTTGTGAGCCAGGGCAT |
| *KLF4 plasmid* | CGCCTTACACATGAAGAGACA | CCTGCACCTGAGGAGTGAAT |
| *LMYCplasmid* | CATACCTCACTGGCTACGGA | CCTGGATTGCTTTCTACATCCC |
| *LIN28plasmid* | AAGAAATCCACAGCCCTACC | CAGCCACCACCTTCTGATAG |
| *EBNA1 plasmid* | CAGGGCCAAGACATAGAGATG | CCACCGTGGGTCCCTTTG |
| *P53plasmid* | CAGTCTACTTCCCGCCATAAA | CAGCCACCACCTTCTGATAG |
| *SOX2gene* | TTCACATGTCCCAGCACTAC | CCCCTCCAGTTCGCTGTC |
| *OCT3/4gene* | CCCCAGGGCCCCATTTTGGTACC | ACCTCAGTTTGAATGCATGGGAGAGC |
| *KLF4gene* | ACCCATCCTTCCTGCCCGATCAGA | TTGGTAATGGAGCGGCGGGACTTG |
| *LMYCgene* | GCGAACCCAAGACCCAGGCCTGCTCC | CAGGGGGTCTGCTCGCACCGTGATG |
| *LIN28gene* | AGCCATATGGTAGCCTCATGTC | TCAATTCTGTGCCTCCGGGAG |
| *GAPDH* | ACCACAGTCCATGCCATCAC | TCCACCACCCTGTTGCTGTA |
| **Sanger sequencing** | | |
| *PROM1 Seq* | CCACCAGGAACAATGCAAACC | CACCTGTTCTCACGCCTAATC |
| **Gene editing** | | |
| *ALELO40RE* | CCTCATCGTAATATTTTACTACTTA | CGTTAAGCTGGTCTAAAATG |
