# Supplementary figures and images for: Clinical exome analysis and targeted gene repair of the c.1354dupT variant in iPSC lines from patients with PROM1-related retinopathies exhibiting diverse phenotypes
Source: Stem Cell Res Ther. 2024 Jul 2;15:192. doi: 10.1186/s13287-024-03804-2 (PMC11218195; doi:10.1186/s13287-024-03804-2)

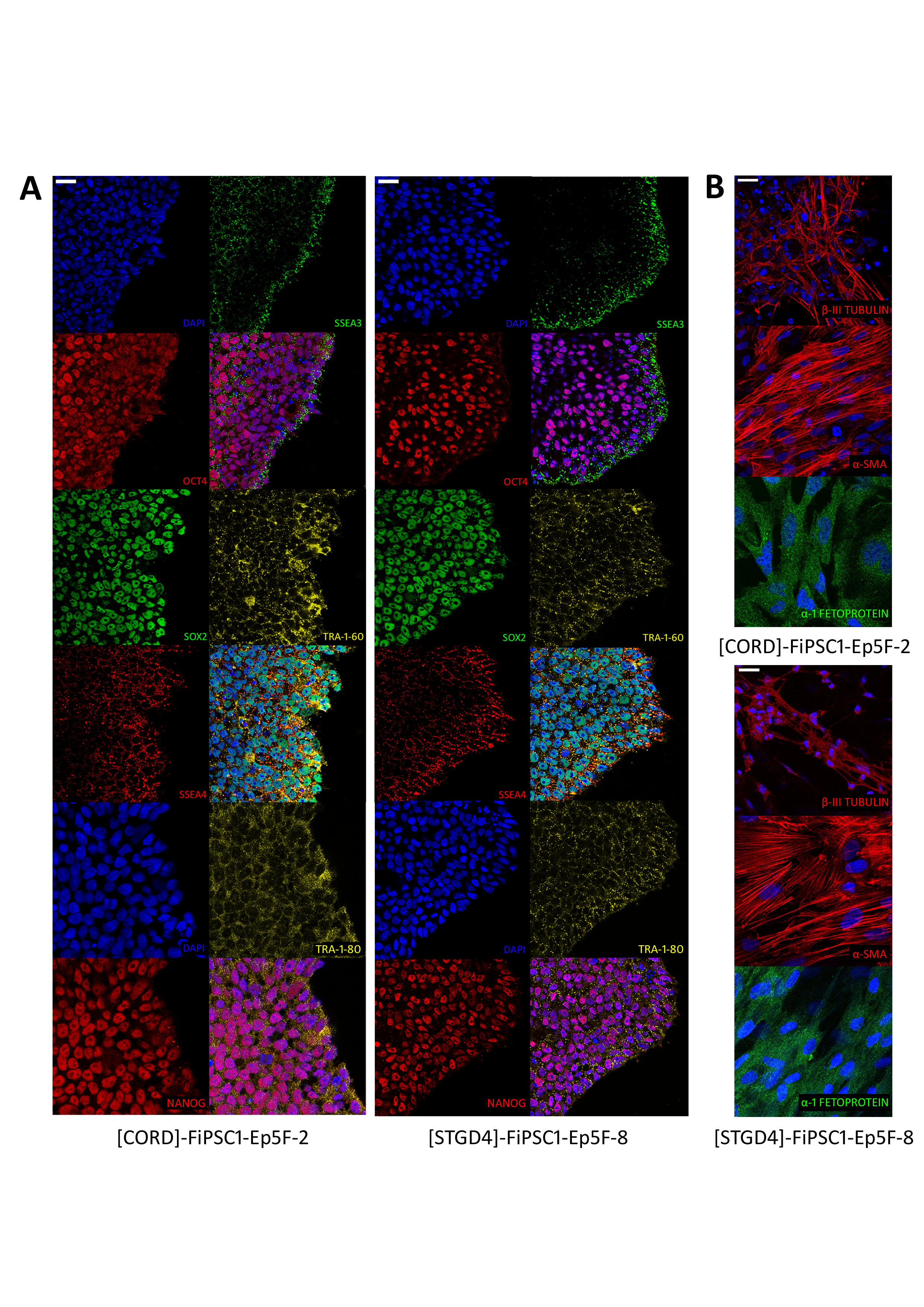

Supplement: Supplementary file 4 — Pluripotency analysis. (A) Immunoreactivity of [CORD]-FiPSC1-Ep5F-2 and [STGD4]-FiPSC1-Ep5F-8 for the pluripotency markers OCT4, SSEA3, SOX2, SSEA4, TRA1-60, NANOG, and TRA-1-81. 25 µm scale. (B) Analysis of the functional pluripotency of the embryoid bodies (EBs) derived from [CORD]-FiPSC1-Ep5F-2 and [STGD4]-FiPSC1-Ep5F-8. Immunoreactivity to ectoderm (β-III Tubulin), mesoderm (α-SMA) and endoderm (α-1 Fetoprotein) markers. 25 µm scale. [file 13287_2024_3804_MOESM4_ESM.tif]

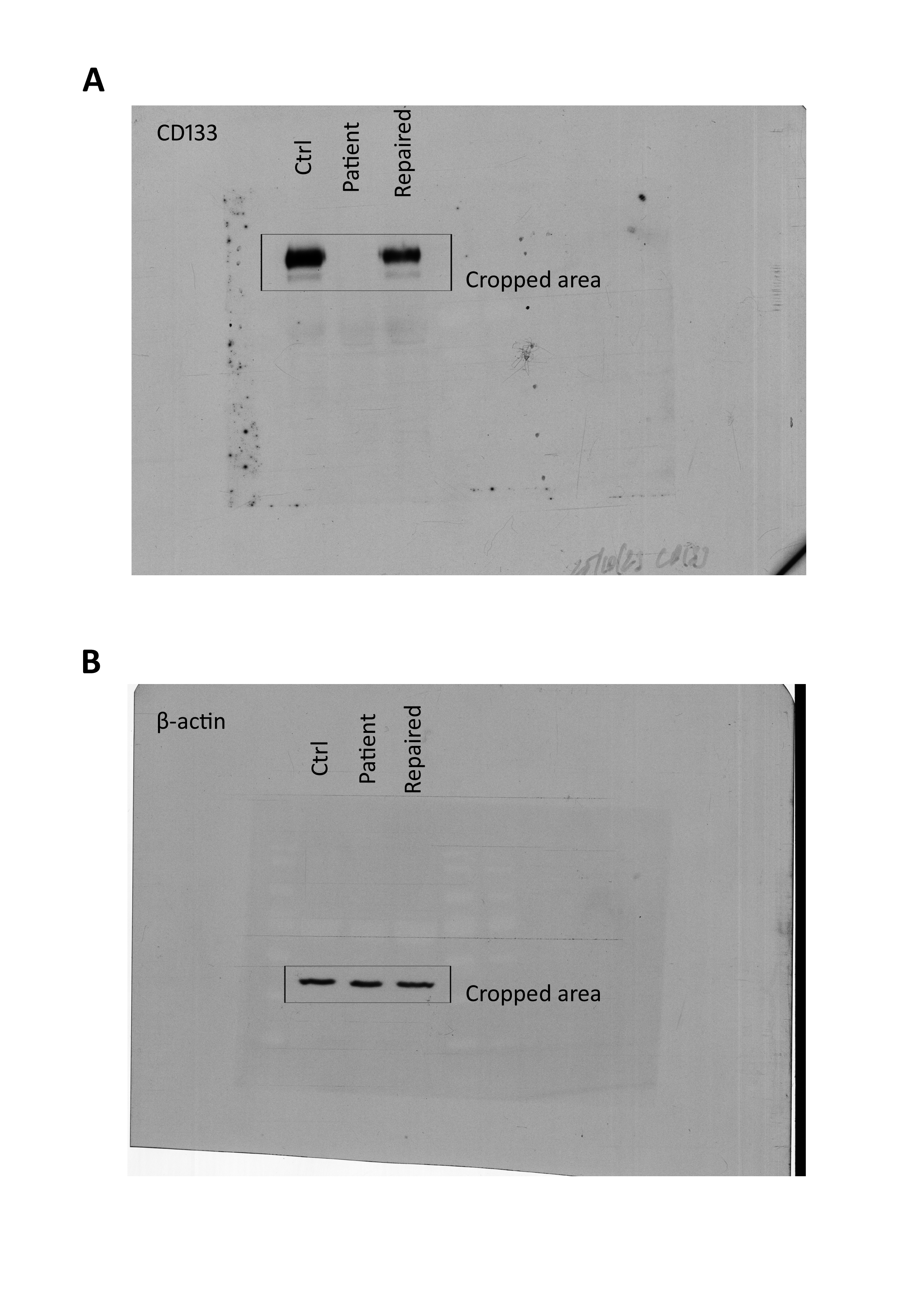

Supplement: Supplementary file 5 — Full-length gels of Figure 4. (A) Full-length gels of Western blotting of CD133 protein expression in the iPSC control line [FiPS] Ctrl1-Ep6F-5, the iPSC line derived from patient IRD2 [RP]-FiPSC1-Ep5F-10, and its corresponding genetically repaired iPSC line [RP]-FiPSC1-Ep5F-10-GC1. (B). Full-length gels of Western blotting of β-actin (control). [file 13287_2024_3804_MOESM5_ESM.tif]
